# Gene Transcription as a Therapeutic Target in Leukemia

**DOI:** 10.3390/ijms22147340

**Published:** 2021-07-08

**Authors:** Alvina I. Khamidullina, Ekaterina A. Varlamova, Nour Alhuda Hammoud, Margarita A. Yastrebova, Alexandra V. Bruter

**Affiliations:** 1 Institute of Gene Biology, Russian Academy of Sciences, 34/5 Vavilov Street, 119334 Moscow, Russia; katerinavarlamova196@gmail.com (E.A.V.); ritayastrebova@genebiology.ru (M.A.Y.); aleabruter@gmail.com (A.V.B.); 2 Moscow Institute of Physics and Technology, 9 Institutskiy Pereulok, 141701 Dolgoprudny, Russia; noor.hammoud@mail.ru

**Keywords:** transcription, leukemia, targeted antitumor therapy, cell death, drug design

## Abstract

Blood malignancies often arise from undifferentiated hematopoietic stem cells or partially differentiated stem-like cells. A tight balance of multipotency and differentiation, cell division, and quiescence underlying normal hematopoiesis requires a special program governed by the transcriptional machinery. Acquisition of drug resistance by tumor cells also involves reprogramming of their transcriptional landscape. Limiting tumor cell plasticity by disabling reprogramming of the gene transcription is a promising strategy for improvement of treatment outcomes. Herein, we review the molecular mechanisms of action of transcription-targeted drugs in hematological malignancies (largely in leukemia) with particular respect to the results of clinical trials.

## 1. Introduction

For a long time, gene transcription has been considered non-druggable because of inevitable general toxicity. However, gene transcription in tumor cells has unique patterns (reviewed in [1]). Malignant transformation, maintenance of tumor cell stemness [2], and other events associated with adaptation of tumor cells to environment largely depend on specific transcriptional patterns [1,3,4,5]. This allows designing anticancer drugs that target gene transcription.

Aberrant activation of signaling pathways is common in tumors. Moreover, leukemias are often caused by formation of new chimeric transcription factors (TFs) as a result of chromosome rearrangements [6]. Specific inhibitors of TFs, although difficult to design, can turn out to be effective anticancer drugs.

Super-enhancers (SE) are the cis-acting elements on DNA recognized by different components of the transcriptional machinery. Unlike conventional enhancers, these potent regulatory elements are capable of switching gene transcription in an ‘all-or-nothing’ manner in response to relatively small changes in chromatin modification, TF concentration, etc. Commonly, SEs control highly but not ubiquitously expressed genes, for example, the genes involved in the maintenance of the pluripotent stage and lineage specificity [7]. These traits and the fact that SEs can often be formed de novo [8,9] by tumor driver genes via mutagenesis, genetic rearrangements, and epigenetic alterations provide evidence that SE components can be promising drug targets [7,10].

Two aspects make ‘transcriptional’ drugs suitable for combinations with other chemotherapeutics. First, the transcriptional modulators can synergize with conventional cytotoxic drugs in achieving antitumor efficacy with better tolerance, which is extremely important in children and elderly patients [11,12]. Second, targeting transcriptional machinery can prevent the establishment of drug resistance (NCT04017546, NCT01434316) [13,14,15,16,17,18]. Here, we review the fundamental aspects related to the design of tools for targeting gene transcription in hematological malignancies and analyze the current state-of-the-art in clinical trials.

## 2. Epigenetics: General Considerations

Chromatin regulators have been identified as drivers of transformation in various blood malignancies. Chromosomal rearrangements (e.g., *MLL* rearrangements (*MLL*-r) in acute myelogenous leukemia (AML) or *BCR–ABL1* in chronic myelogenous leukemia (CML)) and mutations (point mutations in *EZH2* in acute lymphocytic leukemia (ALL)) can affect chromatin state [19,20,21,22,23] and/or activity of the enzymes involved in methylation/demethylation or acetylation/deacetylation of chromatin. These modifications are important for the activation or suppression of transcription (Table 1). This opens the room for development of drugs aimed at restoring epigenetic regulation in leukemias.

### 2.1. Deacetylases: HDACs

Histone deacetylases (HDACs) catalyze the deacetylation of histones at lysine residues, whereas histone acetyltransferases (HATs) perform the opposite function [24,25]. The balanced and controlled activity of HDACs and HATs is required for normal development and homeostasis of hematopoietic cells [25]. Some HATs (such as MYST and the CREBBP/EP300 family) act as transcriptional coactivators together with key hematopoietic TFs and are required for self-renewal and differentiation of HSCs [26]. HDACs are divided into Zn-dependent (classes I, II, and IV) and Zn-independent, NAD-dependent (class III) enzymes [24,26,27,28]. HDACs target the actively transcribed gene regions marked by phosphorylated RNA polymerase II (RNA Pol II) [29]. Among the non-histone targets of HDACs are RNA splicing factors, chaperones, some structural and signaling proteins, TFs, DNA repair proteins, retinoblastoma proteins, and many others [24,28,29]. Interactions of HDACs with non-histone proteins such as Bcl-6 [24,30] and p53 [31] indicate that functions of these proteins are regulated, at least in part, by acetylation.

**Figure 1:**
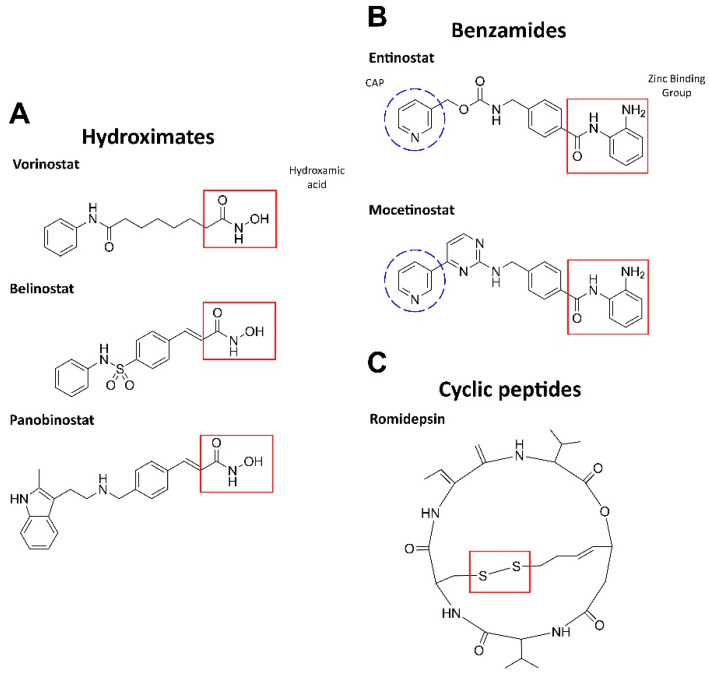
Structures of HDAC inhibitors. (**A**) The active group is hydroxamic acid (red) which binds to the zinc ion in HDAC [24]. (**B**) The zinc-binding moiety is highlighted in red: *ortho*-NH_2_ group and the carbonyl oxygen chelate Zn. CAP is a hydrophobic group for protein surface recognition [34]. (**C**) The disulfide bond in romidepsin is reduced by glutathione to form an active compound. The reduced romidepsin forms a covalent disulfide bond with the sole cysteine residue in the HDAC pocket [35]. The structures were designed by ChemDraw®, a product of PerkinElmer.

HDAC inhibition leads to an increase in the amounts of acetylated histones, which, in turn, promotes the resumption of expression of muted genes and induces differentiation, arrest, and/or death in AML cells. HDAC inhibitors can be either isoform-selective or act against all types of HDACs [32]. Based on the chemical structure and activity for blood malignancies, HDAC inhibitors are largely presented by three groups: hydroximates, benzamides, and cyclic tetrapeptides (Figure 1) [33].

The hydroxamic acid residue directly binds to Zn^2+^ in the active site via the sulfhydryl group. These inhibitors are most potent against class I and II HDACs. Vorinostat (SAHA), panobinostat (LBH589), and belinostat (Beleodaq or PXD101) have been clinically tested in combination with antileukemic agents (NCT00097929, NCT01083602, NCT01273155) but demonstrated limited efficacy (see [24] for a detailed review).

Benzamides (aminoanilides) are the selective class I and IV HDAC inhibitors that bind Zn^2+^ in the active sites [36]. Mocetinostat (MGCD0103) has antiproliferative potency against hematologic malignancies (*MLL*-r leukemia). This agent induces TNF-α, activates NF-κB, regulates JAK/STAT signaling components, downregulates CD30 receptor expression, and reduces lysine specific demethylase 1 (LSD1; see Section 2.3), thereby promoting cell death [33,37]. Phase II clinical trials have been completed for mocetinostatin combination with 5-azacitidine in myelodysplastic syndrome (MDS) or AML patients and demonstrated an acceptable safety profile (NCT00324220) [38]. Entinostat (MS-275) induces growth arrest and apoptosis in AML cell lines and patient samples by inhibiting the antiapoptotic proteins Bcl-2 and Mcl-1, as well as via p21 increase. Moreover, entinostat induced degradation of Fms-like tyrosine kinase 3 (FLT3) through inhibition of Hsp90 chaperone activity in AML cells, suggesting that this inhibitor may be useful for treating patients with FLT3 mutations, a common genetic alteration in AML (reviewed in [24]).

Cyclic peptides are a structurally diverse group of HDAC inhibitors; their selectivity against HDACs depends on the structure [24]. One of the most propitious inhibitors, romidepsin(FK228), is a prodrug that is activated by intracellular reduction to a thiol-containing metabolite and chelates Zn^2+^ in the active site of class I and II HDACs. Moreover, romidepsin activates the stress-activated protein kinases (SAPKs)/Jun amino-terminal kinases (JNKs), as well as inhibits PI3K–AKT–mTOR and Wnt/β-catenin pathways [39]. After the completion of phase II, romidepsin was approved by the FDA for T-cell lymphoma (NCT00106431, NCT00426764), showing an acceptable safety profile [40].

### 2.2. Histoneacetyltransferase: BET

Families of bromodomain (BRD) and extraterminal (BET) proteins BRD2, 3, and 4, recognize histone acetyl-lysine residues, creating the basis for the assembly of protein complexes that regulate the availability of chromatin for TFs and recruitment of RNA Pol II [25,41,42,43,44,45]. BET proteins maintain aberrant chromatin states in AML, ALL, multiple myeloma, and lymphoma [42,43,44]. BRD4 is the most studied member of the BET family. It binds preferentially to the acetylated histones 3 and 4 (H3 and H4) (Table 1). BRD4 marks the transcription start sites of many genes and promotes cell-cycle progression from G1 to S and from G2 to mitosis. BET proteins play a central role in tumorigenesis associated with TFs of the MYC family [46]. BET inhibitors suppress SE-associated oncogenes and block tumor cell proliferation [21,42,45,46,47,48]. These compounds reduce *MYC* expression in AML cells sensitive or resistant to BET inhibition. However, the resistant leukemias showed a rapid return of *MYC* transcription [48,49,50]. Most BET inhibitors cause G1/S arrest [48,50,51,52].

JQ1, a selective BRD2/4 inhibitor, inhibits the binding of the Mediator–BRD4 complex to acetylated histone residues. JQ1 can selectively repress *MYC* transcription in blood malignancies [53,54,55] and is active against MLL3-suppressed leukemias resistant to conventional chemotherapy [53,56]. BRD2 is a critical mediator of STAT5 function. This TF is constitutively active in most leukemias and controls the expression of genes involved in cell proliferation and survival (see Section 4.5) [45]. JQ1 treatment reduced STAT5-dependent transcription and showed a strong synergy with tyrosine kinase inhibitors in inducing apoptosis in leukemic cells [45,57]. The main drawback of JQ1 is its short half-life (~1 h) [58]. OTX015 (birabresib), an analogue of JQ1, is more stable [51] and inhibits the binding of BRD2–4 to acetylated H4 (IC_50_ < 200 nM for AML and ALL cell lines [51]). OTX015 completed phase I clinical trials for AML, diffuse large B-cell lymphoma, ALL, and multiple myeloma with promising prospects (in particular, relatively low dose-limiting toxicity) (NCT01713582). I-BET762 (GSK525762) and I-BET-151 (GSK1210151A) (100-300 nM) evoked an antiproliferative effect associated with suppression of *BCL2* and *CDK6* genes in AML cells including drug resistant counterparts [48,56]. I-BET762 has completed phase II of clinical trials (NCT01943851). BI 894999 is a selective BET inhibitor that causes apoptosis in the AML cell line MV4-11B at 10 nM [50]. Using RNA sequencing, it has been shown that BI 894999 and JQ1 regulate the same transcripts, including *MYC* [50].

### 2.3. Histone Demethylase: LSD1

Lysine-specific histone demethylase 1A (LSD1, also known as lysine KDM1A, AOF2, BHC110) is a FAD-dependent histone demethylase often overexpressed in lymphoid malignancies. LSD1 contributes to leukemogenesis in ~60% of AML cases [59,60,61] by delaying the maturation and promoting the proliferation of myeloid precursors [60]. LSD1 can be a component of the NuRD (nucleosome remodeling and deacetylase) complex, which has a function of nucleosome remodeling via histone deacetylase/demethylases activities and is recruited to cell type-specific SEs [61]. LSD1 interacts with the TF corepressor RE1 (CoREST, RCOR1) and HDAC1-2 [37,61,62]. LSD1 demethylates mono- and dimethyl groups at H3K4 (H3K4me1/2) and H3K9 (H3K9me1/2) (Table 1), as well as several non-histone targets [60,62,63,64,65]. H3K4me1 and H3K27ac are the markers of enhancer activation [66]; therefore, LSD1 functions to repress the enhancers. In murine hematopoietic cells, the loss of LSD1 causes pancytopenia associated with activation of genes previously repressed by LSD1 and elevation of H3K27ac at the enhancers of LSD1 target genes [67].

RUNX1 (Runt-related TF 1, also known as the AML protein 1 and the core binding factor subunit alpha-2, CBFA2) interacts with the LSD1–CoREST–HDAC1/2 complex which, together with GFI1B (growth factor independent 1B transcriptional repressor), suppresses myeloid differentiation in HEL (erythroleukemia) and MEL (lymphoma) cells [62]. RUNX1 regulates the expression of proteins associated with hematopoiesis (e.g., C/EBPα and PU.1) or cell cycle (e.g., p53). A conditional *RUNX1* knockout causes thrombocytopenia and lymphocytopenia [12]. PU.1 is a TF that is specifically expressed in myeloid cells and B-lymphocytes, thereby activating the genes involved in differentiation of these cells [12]. Inhibition of LSD1 caused an increase in chromatin availability with strong enrichment in PU.1, C/EBPα, and RUNX1, whereas the loss of C/EBPα or PU.1 led to the resistance of AML cells to LSD1 inhibition both in vitro and in vivo, showing the importance of PU.1 and C/EBPα in modulating the antileukemic efficacy of LSD1 inhibition [59,60,68]. Mutations associated with the loss of RUNX1 and C/EBPα function result in a high risk of AML often associated with complex karyotype and resistance to chemotherapy [69].

Trianylcypromine (TCP) is the main scaffold in the design of irreversible LSD1 inhibitors. TCP-based LSD1 inhibitors include ORY-1001, GSK2879552, and IMG-7289 that are undergoing clinical trials alone or in combination with all-*trans* retinoic acid (ATRA) for AML [63]. ORY-1001 binds covalently to FAD in complex with LSD1 [70,71]. ORY-1001 induced myeloid differentiation and cytotoxicity in AML and CML cell lines (IC_50_= 0.05–0.4 nM) [72]. ORY-1001 synergizes with conventional drugs ATRA and Ara-C and targeted inhibitors in AML and ALL cell lines [72]. ORY1001 is currently in phase I of pharmacokinetic and safety studies for patients with relapsed or refractory AML (EudraCT Number: 2013-002447-29). Another covalent LSD1 inhibitor GSK2879552 [70] increased the expression of myeloid differentiation markers *CD11B* and *CD86* [73] but did not induce noticeable caspase 3/7 activation, which implies that the cytotoxic effect of the LSD1 inhibitors is due to the impairment of cell division [73]. GSK2879552 inhibited cell proliferation with an average IC_50_= 137 nM in 20 cell lines [73] via G0/G1 arrest [59]. Phase I of the GSK2879552 study in AML patients was discontinued because of the high risk of relapse (NCT02177812). The irreversible inhibitor IMG-7289 selectively suppressed proliferation and induced apoptosis of JAK2^V617F^ cells (a mutation in Janus kinase 2 that triggers a constitutive activation of the JAK–STAT pathway [74]) due to simultaneous increase in the expression and methylation of p53 and, independently, of the proapoptotic protein PUMA, as well as by reducing the antiapoptotic BCL-xL [75]. IMG-7289 is currently being tested in multiple phase I/II trials for different blood malignancies (NCT03136185, NCT04254978, NCT04262141, NCT03136185, NCT04081220).

In addition to TCP derivatives, studies are underway to identify reversible LSD1 inhibitors. SP-2509 is a highly selective LSD1 inhibitor that binds to the FAD pocket [76]. SP-2509 increased the levels of dimethylated and trimethylated H3K4 associated with the promoters of genes targeted by LSD1, as well as the levels of p53, p21, and C/EBPα in AML cells [76]. Furthermore, NCD25 and NCD38, the low-molecular-weight LSD1 inhibitors, were potent against AML and CML cell lines at nanomolar concentrations [61]. NCD38 induces transdifferentiation from the erythroid to the granulomonocytic lineage with derepression of ~500 SEs in HEL cells [62]. NCD38 selectively disrupts the interaction between LSD1 and GFI1B, which increases the level of H3K27ac on specific SEs [62]. This activates the genes associated with myeloid differentiation such as *GFI1, CEBPA, CEBPE, CLEC4A*, and *MPO* [61], induces *ERG* (Ets-related gene), and increases the expression of *C/EBPα, PU.1*, and *RUNX1* [62]. Epiberberine is a natural product [77] that reversibly inhibits LSD1 [70]. Epiberberine suppresses the proliferation of leukemia cells by increasing H3K4me2 and H3K9me2, especially in the *CD11D* and *CD14* gene regions (markers of myeloid differentiation) [70].

### 2.4. Histone Methyltranferases

#### 2.4.1. MLL

The enzyme whose name derives from mixed-lineage leukemia (MLL, also known as MLL1, KMT2A, etc.) is one of six histone methyltransferases (HMTs) of the mixed-origin leukemia family [78,79,80]. MLL catalyzes mono-, di-, and trimethylation of H3K4 (Table 1) via the evolutionarily conserved SET domain. Both MLL and H3K4me are localized to gene promoters close to transcription initiation sites. There are ~50 genes specifically regulated by MLL, including *HOXA9*, *MYC*, and *BCL2*. Dysregulation of MLL1 accounts for 5–10% of AML cases in adults and almost 70% of childhood ALL [78,79].

MLL has an extremely low HMT activity by itself. This property is sharply increased when MLL is assembled into complexes with WDR5, ASH2L, and RbBP5 [81]. All three proteins contribute to optimal activity of the MLL complex, although the mechanism is different. Depletion of any of these proteins leads to a sharp decrease in the overall activity of the complex. Importantly, the interaction between MLL and WDR5 is critical for the integrity of the MLL complex and, hence, its methyltransferase activity [78]. The most common *MLL*-r are translocations that lead to formation of the oncogenic MLL fusion proteins. These aspects are analyzed in Section 4.3.

The inhibitor MM-401 selectively represses MLL activity by blocking the assembly of the MLL1–WDR5 complex, whereas other MLLs remain unaffected. MM-401 specifically blocks the proliferation of MLL–AF9, MLL–ENL, and MLL–AF1 cells, causing an arrest in G1/S, apoptosis, and myeloid differentiation without damage of the bone marrow or other normal cells [78].

#### 2.4.2. G9a

The euchromatic histone lysine methyltransferase 2 (EHMT2, also known as G9A/KMT1C) and GLP (G9a-like protein, also known as EHMT1 or KMT1D) have 80% sequence identity in their catalytic domains, form homo- and heterodimers, and catalyze mono- and dimethylation of H3K9 (Table 1) [82,83]. G9a and GLP are also capable of methylating H3K27, lysine 373 in p53, and other regulators of gene expression [83,84]. G9a induces changes in cellular redox homeostasis, which leads to a decrease in the production of reactive oxygen species (ROS) [82] and can partially activate transcription by acting as a cofactor for the Mediator complex [85]. High levels of G9 expression are associated with adverse clinical outcomes including metastasis and treatment resistance [82,84]. Pharmacological and genetic suppression of G9a has been shown to effectively slow down the proliferation of AML cells and leukemia stem cells (LSCs) in a mouse model due to suppression of HOXA9-dependent transcription [84,85]. The G9a inhibitor BIX-01294 induced apoptosis in AML cell lines. However, the effect in LSC-like KG-1 cells was limited [82]. PKR-like endoplasmic reticulum kinase (PERK) limits the accumulation of ROS through phosphorylation and stabilization of NRF2 (nuclear factor erythroid 2-related factor 2), increasing the synthesis of glutathione, and increasing the regulation of heme oxygenase-1 (HO-1) [86]. Inhibition of G9a led to activation of the PERK/NRF2 pathway and upregulation of HO-1 in KG-1 cells, while treatment with a PERK inhibitor enhanced caspase-independent apoptosis, indicating that PERK/NRF2 may be a marker of resistance to G9a inhibitors [82]. At the same time, there is no significant proapoptotic effect on normal HSCs when combining BIX-01294 with the PERK inhibitor [82]. The inhibitor DCG066, unlike BIX-01294, induced apoptosis due to retardation of K562 cells (CML) in the G2/M phase, although BIX-01294 and DCG066 form hydrophobic contacts with similar amino-acid residues in the peptide substrate pocket [84]. A-366 is a peptide molecule that selectively inhibits G9a/GLP and moderately inhibits the AML MV4-11 xenograft model [83].

#### 2.4.3. EZH1/2

Enhancer of zeste homologs 1 and 2 (EZH1 and EZH2) are catalytic subunits of the Polycomb repressive complex 2 (PRC2) responsible for transcriptional repression via trimethylation of H3K27 (Table 1) [23,65,87]. EZH1 and EZH2 are antagonists of the BAF complex (see Section 2.5.2). PRC2, together with embryonic ectoderm development (EED) and suppressor of zeste 12 (SUZ12), regulates cell lineage determination and homeostasis [88], supporting the multipotency and self-renewal of HSCs [65]. Deletions and point mutations of *EED*, *EZH2*, and *SUZ12* are found in 42% of early T-cell precursor (ETP) ALL cases, as well as in 12% of T-cell ALL cases in non-ETP malignancies [23]. Overexpression of EZH2 is associated with the development of hematopoietic malignancies [89]. EZH2 point mutations in tyrosine 641 (Y641F, Y641N, Y641S, and Y641H) have been identified in 8–24% of lymphomas [89], whereas mutations A677G and A687V have been identified in non-Hodgkin’s lymphomas [90,91]. All of these genetic alterations increased the enzyme activity and elevated the levels of H3K27me3.

3-Deazaneplanocin A (DZNep) inhibits *S*-adenosyl homocysteine hydrolase which disrupts methionine metabolism leading to inhibition of methyltransferases. Suppression of EZH2 by DZNep has been shown to inhibit the formation of leukemia colonies and reduce H3K27me3 [92]. However, DZNep has a very short half-life in the plasma; this compound induces nonspecific inhibition of histone methylation and is toxic in animal models [23]. Inhibitors EPZ005687, GSK126, and EI1 competitively bind to *S*-adenosylmethionine (SAM), a substrate for normal and mutant EZH2, and inhibit the enzyme > 50 times more selectively than EZH1. All three inhibitors showed activity against lymphoma cells with EZH2 mutation [89,93,94].

Tazemetostat (EPZ-6438) is more potent than EPZ005687 and has better pharmacokinetic properties, including oral bioavailability [95]. Tazemetostat is undergoing phase I/II clinical trials against B-cell lymphomas (NCT01897571) with favorable results; sustained responses were observed in 38% of patients with non-Hodgkin B-cell lymphoma [96].

Double EZH1/2 inhibition increased the efficacy against MLL leukemia compared to single-isoform inhibition. For example, UNC1999 [87] and SAH-EZH2 peptide (binds EED, resulting in dissociation of EZH1–EED and EZH2–EED complexes) showed activity against MLL leukemia [88]. SAH-EZH2 causes growth arrest and differentiation of MLL-AF9 cells with little effect on normal hematopoietic cells [88].

#### 2.4.4. DOT1L

DOT1L is a methyltransferase that catalyzes mono-, di-, and trimethylation of H3K79 (Table 1) [65,97,98,99], which is necessary for G1/S transition [100]. Aberrant DOT1L recruitment is associated with an abnormally high H3K79me2 abundance on the promoters and bodies of MLL target genes in the *MLL*-r leukemia [65,97]. Inactivation of DOT1L significantly suppressed the *HOXA9* and *MEIS1* genes associated with *MLL* translocation and leukemogenesis, leading to decreased proliferation, increased differentiation, and apoptosis of MLL–AF9 cells, indicating its potential for AML therapy [37,65,97,99]. MLL fusions with proteins interacting with DOT1L (e.g., AF9, ENL, AF17, and AF10) incorrectly target DOT1L to the promoters of the *HOXA* genes, which leads to methylation of H3K79 and constitutive activation of these genes [22,98,101,102]. This makes DOT1L methyltransferase a propitious therapeutic target in leukemia.

EPZ4777 is a selective DOT1L inhibitor [103]. The high level of H3K79 methylation correlates with the increased expression of genes of the *HOXA* and *MEIS1* cluster [60]. EPZ4777 specifically inhibits H3K79 methylation and mediates the suppression of these genes, which results in chromatin inaccessibility in the *HOXA* and *MEIS1* gene regions [59]. Pinometostat (EPZ-5676), a low-molecular-weight inhibitor of DOT1L, caused an antiproliferative effect at submicromolar concentrations [71,99,104] and is currently being examined in phase II clinical trials for AML (NCT03724084). Pinometostat selectively inhibits methylation of H3K79, resulting in a decreased transcription of the MLL target genes *HOXA9* and *MEIS1* [105]. Pinometostat also decreases the expression of the *KDM1A* (LSD1 protein-coding gene) which may indicate a relationship between HOXA9 and LSD1 [37]. Resistance to pinometostat tested MLL cell lines emerged 3weeks post treatment through various mechanisms, including activation of PI3K/AKT and RAS/RAF/MEK/ERK and overexpression of drug efflux transporter ABCB1 [99].

#### 2.4.5. PRMT

Arginine methylation is catalyzed by arginine methyltransferases (PRMTs) classified into types I and II. All PRMTs catalyze the formation of a monomethylated intermediate, and type I PRMTs (PRMT1, 2, 3, 4, 6, and 8) additionally catalyze the production of asymmetrically dimethylated H4 while the type II PRMTs (PRMT5 and 9) generate dimethylated H4 [106]. PRMT1 and PRMT5 are extensively studied in relation to their action against leukemia [107]. PRMT1 promotes methylation of H3R4 which is associated with the active state of chromatin on the promoters critical for differentiation of hematopoietic cells [65]. PRMT1 is the most abundant arginine methyltransferase in mammalian cells that acts as a transcriptional coactivator and regulates numerous cellular processes including DNA damage and cell cycle [108]. PRMT1 is significantly increased in AML cells compared to normal hematopoietic counterparts [109]. Inhibition of PRMT1 blocks MLL–GAS7- and MLL–EEN-driven leukemogenesis [110].

### 2.5. Other Chromatin Modulators

#### 2.5.1. KAT2A

KAT2A is a histone acetyltransferase whose inhibition stimulates LSCs to differentiate via stabilization of gene expression programs [111]. An increased *KAT2A* expression has been found in children with AML [112]. KAT2A exhibits its acetyltransferase activity in the context of two macromolecular complexes, Spt-Ada-Gcn5-acetyltransferase (SAGA) and Ada-Two-A-Consing (ATAC) [111]. Both complexes contribute to the spread of leukemia and affect certain aspects of metabolism and proliferation (ATAC), as well as cell identity and survival (SAGA), together explaining the need for KAT2A in LSCs [113]. KAT2A inhibition induces differentiation and apoptosis in AML cells but not in normal progenitors [114], thereby serving as a promising avenue for drug development.

#### 2.5.2. CREBBP/EP300

The lysine acetyltransferase paralogs CREBBP (CBP, KAT3A) and EP300 (KAT3B) are transcriptional coactivators that regulate many cellular processes [65]. SEs are enriched in CREBBP/EP300 compared to conventional enhancers, and EP300 recruits BRD4 in mouse leukemia cells [25]. CREBBP and EP300 can merge with monocytic leukemia zinc finger protein (MOZ) or MLL [65]. Inhibition of EP300/CREBBP bromodomains mediates antiproliferative responses in AML and CML cell lines by interfering with transcription of oncogenes such as *MYC* [115] and inducing G0/G1 arrest [25]. GATA1/MYC is a key component of the mechanism of action of the EP300/CREBBP bromodomain inhibitors in the K562 cell line [25].

#### 2.5.3. BAF

The ATP-dependent BAF chromatin-remodeling complex (SWI/SNF complex in yeast, BAP in *Drosophila melanogaster*) is critical for the regulation of gene expression and differentiation [116,117]. As part of a large (~2 MDa) complex containing >15 subunits, BAF activates gene expression which is thought to result from its capacity to remodel and evict nucleosomes at gene promoters [118]. The evolutionarily conserved BAF complex contains one of two closely related ATPases, BRM or BRG1, and uses the energy of ATP hydrolysis to remodel the chromatin structure. It has been shown in vitro and in vivo that the enzymes of the complex can facilitate the binding of transcription activators and TATA box binders, as well as support the formation of preinitiation and elongation complexes associated with RNA Pol II [119]. BAF subunits, BRG1 and INI1, bind in vivo to *RUNX1*-regulated promoters (e.g., *GMCSF, IL3, MCSF-R, MIP*, and *CDKN1A*). These interactions are associated with chromatin remodeling during myeloid differentiation [119]. BAF mutations have been detected in 20% of all cancers [3]. In lymphoma, mutations in the BAF complex lead to a noticeable transcriptional heterogeneity within the tumor [3,118]. The complex is critical for the proliferation and viability of leukemic cells [8]. BAF is involved in the formation of ALL resistance to glucocorticoids [120]. Inhibition of the bromodomain containing BRD9, a subunit of the BAF complex, showed antileukemic activity in the AML model (MOLM-13 line) [121]. These data indicate the attractiveness of the BAF complex as a target for antileukemia therapy.

The research in the field of drugs targeted at epigenetic regulators is actively developing. Current clinical trials look promising for these approaches be incorporated into the routine therapy of blood malignancies (Table 2).

## 3. Transcription-Associated Cyclin-Dependent Protein Kinases

Cyclin-dependent kinases (CDKs) represent a family of 20 serine/threonine kinases that control critical cellular processes. CDKs are divided into two groups: cell-cycle regulators (including CDK1, CDK2, CDK4, and CDK6) and transcription regulators (CDK7, CDK8/19, CDK9, CDK12, and CDK13). The first group controls the transition between cell-cycle phases, while the second group regulates gene transcription. Transcription-associated CDKs are recruited to chromatin as parts of much larger complexes. Upon binding to their cyclin partners, CDKs phosphorylate serine 2, 5, and 7 in the CTD of RNA Pol II, leading to transcription initiation and elongation [122,123]. Some CDKs regulate both processes, for example, CDK7 drives cell-cycle progression, in addition to its role in transcription [124]. Important roles of CDKs in proliferation and gene expression, as well astheir deregulation in many cancers, have revealed potential therapeutic opportunities opened by targeting these kinases [122]. Numerous studies have implicated CDKs in tumorigenesis, particularly in myeloproliferative neoplasms (MPNs) [125,126,127]. AML is characterized by disruption in gene transcription and aberrant expression of transcriptional CDKs. Various novel CDK inhibitors have been developed for AML treatment [128]; some of them are analyzed below. It is worth noting that, although promising, low-molecular-weight inhibitors of transcription-associated CDKs have not yet entered the conventional treatment protocols [123].

### 3.1. CDK7 and CDK9

CDK7 and CDK9 are considered key regulators of the transcriptional machinery. In a simplified model, CDK7, a component of the TFIIH (transcription factor IIH) complex, phosphorylates Ser5 and Ser7 at CTD of RNA Pol II, leading to initiation of transcription. CDK9 is the catalytic subunit of the P-TEFb complex, and CDK9 phosphorylates Ser2 at CTD of RNA Pol II, enabling transcriptional elongation. However, functions of both CDKs are context-specific, and these kinases are, to some extent, complementary and interchangeable [122]. AML often harbors mutations in genes responsible for transcription, which make them susceptible to CDK7 inhibition [129,130]. In addition, deregulation of the CDK9 pathway has been linked to AML and other hematologic malignancies [131]. These findings suggest that targeting CDK7 and CDK9 in AML may be a viable therapeutic option.

Pan-CDK inhibitors alvocidib and seliciclib can be used to combat AML. Alvocidib (formerly flavopiridol) is a CDK1, 2, 4, 6, 7, and 9 inhibitor with a pronounced potency against CDK9. Seliciclib ((*R*)-roscovitine) is active against CDK1, 2, 5, 7, and 9. Primary effects of alvocidib and seliciclib include CDK9 inhibition, thereby suppressing SE transcriptional targets and activating apoptosis due to the loss of myeloid cell leukemia-1 (Mcl-1) protein. Mcl-1 is a member of the Bcl-2 family of proteins involved in the progression and survival of AML cells as well as chemotherapeutic drug resistance. A high *MCL1* expression in tumor cells is mediated by SEs [132,133].

Dinaciclib is a potent inhibitor of CDKs 1, 2, 5, and 9 and with a higher selectivity and better safety profile than flavopiridol. Dinaciclib may be used to resolve antitumor drug resistance in *MLL*-r AML. The effect of dinaciclib in MLL-driven AML is mediated in part by CDK9 inhibition and involves a decreased expression of the survival protein Mcl-1 [13].

Early CDK inhibitors including alvocidib and dinaciclib have been tested against a number of leukemias. A phase II clinical study of alvocidib is currently underway in patients with refractory/relapsed AML after venetoclax and azacytidine or decitabine combination therapy (NCT03969420). However, while these inhibitors have demonstrated a robust response in AML trials, their clinical utility is limited due to a low selectivity for CDK9 and other CDKs [134]. This led to the development of compound JSH-009 with enhanced selectivity toward CDK9 and favorable pharmacokinetic properties. JSH-009 downregulated *MCL1* and *MYC* mRNAs and proteins and showed a potent antitumor efficacy in preclinical AML models [134].

Fadraciclib (CYC065) is a more selective and potent derivative of seliciclib directed against CDK9 and CDK2. Inhibition of CDK9 by fadraciclib attenuates transcription of the *MCL1* and *BCL2* genes. Inhibition of CDK2 sensitizes AML cells to apoptosis [132]. Fadraciclib showed promising results in mouse xenografts of human AML and has recently reached two phase Ib clinical trials in relapsed/refractory AML/MDS and chronic lymphocytic leukemia in combination with Bcl-2 inhibitor venetoclax(NCT04017546).

LDC067 is another highly selective CDK9 inhibitor that abolishes phosphorylation at Ser2 of RNA Pol II and selectively inhibits transcription. Treatment with LDC067 led to apoptosis in several cancer cell lines and AML blasts. The loss of short-lived mRNAs, including *MYC* and *MCL1*, in LDC067 treated cellsis thought to be the cause [122]. According to a study by Gerlach and colleagues, LDC067 synergizes with the BET bromodomain inhibitor, BI 894999, in AML cell lines, resulting in an enhanced tumoricidal effect in vitro and tumor regression in vivo. Nonetheless, the relatively low potency of LDC067 compared to alvocidib emphasizes the need for further optimization [14].

THZ1, a covalent CDK7 inhibitor [135] exhibited antitumor activity against hundreds of tumor cell lines. Moreover, THZ1 turned out to be nontoxic for normal cells. Although CDK7 has functions in transcription and cell cycle, the antitumor effect of CDK7 has been attributed to SE-associated transcriptional modulation [136]. THZ1 was efficient against T-ALL and peripheral T-cell lymphoma models (reviewed in [136,137]).

CT7001 is a novel CDK7 inhibitor. In preclinical models, CT7001 effectively impeded growth of many AML cell lines and led to tumor regression in MV-4-11 xenograft mice [138,139]. Currently, CT7001 is in two phase I/II trials to evaluate safety and tolerance (NCT03363893).

### 3.2. CDK8/19

CDK8 is a part of the kinase module that associates with the Mediator complex. CDK8 kinase module consists of four subunits: CDK8, MED12, MED13, and cyclin C. CDK8 or its paralog CDK19 regulates transcription by phosphorylating RNA Pol II and TFs (including STAT1, STAT3, STAT5 [140], SREBPs [141], E2F1 [142], and probably others [143]). Numerous studies have identified CDK8 as a critical and unique mechanism of gene regulation in a variety of cancers [47,140,144]. In hematological malignancies, CDK8/19-mediated suppression of SE-associated genes contributes to tumorigenesis. Due to its important role in pluripotent stem-cell development, CDK8 knockout in embryonic stem cells impedes embryonic development. Nevertheless, CDK8 depletion has no effect on normal cell growth, and this fact makes it an extremely attractive drug target [123,126].

The natural antiangiogenic alkaloid Cortistatin A (CA) is extremely potent and relatively selective CDK8/19 inhibitor. Dual CDK8/19 inhibition by CA showed antiproliferative activity in MV-4-11 and SET-2 AML mice xenograft models [126]. In addition to preventing phosphorylation of RNA Pol II, CA inhibits CDK8-mediated Ser727 phosphorylation of STAT1, a mechanism via which CA impedes growth in multiple leukemic cell lines including MOLM-14 (MLL–AF9 fusion). However, the same study that reported MOLM-14 regression as a result of inhibition of STAT1 phosphorylation indicated that deregulation of SE-driven genes plays a role in MOLM-14 sensitivity to CA therapy [47]. In myeloproliferative neoplasms, CA upregulates SE-associated genes that mediate hematopoiesis, tumor growth inhibition, and differentiation of JAK2-mutant megakaryocytes [145,146]. Since treatment of JAK2-mutant MPN patients with ruxolitinib, a JAK1/2 inhibitor that inhibits STAT1 tyrosine phosphorylation, does not induce differentiation, a combination of CA and ruxolitinib has been proposed as a strategy to treat JAK2-mutant MPNs characterized by STAT1 hyperphosphorylation [147]. Along with CDK8/19, ROCK II kinase and CDK11 are also high-affinity CA targets [148].

Senexin A and Senexin B have been discovered in a high-throughput screening for low-molecular-weight inhibitors of p21-induced transcription [149]. Senexin A and its more potent and selective derivative, Senexin B, have been found to be highly selective CDK8/19 inhibitors. Little is known about the effects of Senexin A and Senexin B in AML. A study by Rzymski and colleagues reported that Senexin B potently suppressed phosphorylation of STAT5 Ser726 and STAT1 Ser727 in AML cell lines KG-1, HL-60, MOLM-16, MV-4-11, OciAML-2, and MOLM-6 [140].

SEL120-34A is a substituted tricyclic benzimidazole that represents a novel, robust, and selective CDK8 inhibitor [140]. SEL120-34A has proven effective in AML cells expressing high levels of STAT5 and STAT1. SEL120-34A showed activity in murine AML xenografts (KG-1 and MV-4-11 derived tumors). SEL120 showed synergy in combination with cytarabine. When combined with venetoclax, SEL120 treatment caused apoptosis in AML cells and total regression in MV4-11 xenograft models [16]. Tumor cell death caused by SEL120-34A was mediated by *MCL1* transcriptional silencing [125]. SEL120-34A is currently under investigation in a first-in-human phase Ib study in AML or high-risk MDS (NCT04021368) [147].

Beyond its kinase activity, CDK8 has been identified as a key mediator of BCR–ABL1-driven leukemia. CDK8 was found to be involved in the PI3K/mTOR signaling pathway, and its loss increased sensitivity to mTOR inhibitors. As a result, combined inhibition of CDK8 and mTOR in AML and ALL was proposed. YKL-06-101 is a dual CDK8 degrader and mTOR inhibitor that was generated by combining THZ4-55, an integrated mTOR/CDK8 inhibitor, with thalidomide, which acts as a degrader. This degradation of CDK8 is crucial for its action in order to eliminate kinase-independent activity of CDK8. In both BCR–ABL1-positive and BCR–ABL1-negative CML and ALL, treatment with YKL-06-10 caused growth arrest and cell death, while other members of the Mediator kinase module remained unchanged [150].

### 3.3. CDK12 and CDK13

CDK12 and CDK13 are closely related CDKs with approximately identical kinase domains but different N- and C-terminal domains. CDK12 and CDK13 exert their kinase activity by binding to cyclin K. CDK12 regulates gene transcription, genomic stability, and DNA damage response, and it is crucially important for embryonic development. CDK13 controls gene transcription as well. There is considerable evidence (obtained using genetic methods) for the roles of CDK12/13 in development of breast, ovarian, colorectal, and pancreatic cancer [151]. However, selective small-molecule inhibitors of CDK12/13 are in a short supply [122]. Fan and colleagues developed an ‘analogue-sensitive’ MV-4-11 AML model and demonstrated that dual inhibition of CDK12 and CDK13 induces cell death [152]. THZ1, a covalent CDK7 inhibitor (see above), suppresses CDK12/13 activity at high concentrations and was used as a lead compound for the synthesis of THZ531, a more selective CDK12/13 inhibitor [153]. Treatment of T-cell ALL with THZ531 decreased CTD RNA Pol II phosphorylation and induced apoptosis. THZ531 also downregulated the expression of DNA damage-related, SE-driven genes in AML and CML cells [153]. One problem of the use of THZ compounds is the fast onset of the ABC transporter-mediated drug resistance. To address this issue, a novel covalent inhibitor E9, which functions independently of ABC transport, has been synthesized [154].

Not only transcriptional CDKs can be regarded as therapeutic targets. Although chemical inhibitors have not yet been developed, there are genetic indications that other members of coactivator complexes can be successfully targeted to treat AML. For example, Xu and colleagues demonstrated that TAF12, a member of the TFIID complex, which also binds to and stabilizes the proto-oncogenic TF MYB, is critical for AML suppression [155].

Transcriptional addiction is a hallmark of AML that could be exploited therapeutically, making transcriptional CDKs plausible targets. Despite the notion that targeting transcription is considered somewhat a risky approach due to non-selectivity toward tumor cells, these highly proliferative cells are more vulnerable to transcriptional inhibitors. Several small-molecule inhibitors of CDKs have already reached clinical studies for blood malignancies, while others are still in preclinical stages (Table 3). Currently, much effort is underway to improve the selectivity and pharmacokinetic properties of CDK inhibitors, and recent advancements are promising.

## 4. Fusion Proteins as Transcriptional Modulators

Chromosomal rearrangements, mainly translocations, and the corresponding gene fusions may lead to tumor initiation or progression. Fusion proteins were the first TFs identified as cancer drivers. Fusion oncogenes are crucial in the diagnosis and treatment of various subtypes of leukemia [6]. Since transcriptionally competent fusion proteins drive the disease, they can be considered as targets for drug discovery. The most common balanced rearrangements (chromosomal aberrations with no loss or gain of genetic material) in AML are t(15;17)(q22;q21) *PML–RARA*, t(8;21)(q22;q22) *RUNX1–RUNX1T1*, t(11;v)(q23;v) *MLL*-r, and inv(16)(p13q22) core-binding factor β (CBFβ)–smooth muscle myosin heavy chain (SMMHC, also known as myosin 11) (*CBFB–MYH11*). The oncogenic fusion receptor tyrosine kinase BCR–ABL1 generated upon t(9;22)(q34;q11) deserves to be mentioned among other fusion proteins because it upregulates several important signaling pathways. Inactivation of the enzymatic activity in the BCR–ABL1 fusion protein with the small molecule imatinib (Gleevec®) cures or leads to remission in BCR–ABL1-positive malignancies [156]. However, targeting fusion oncoproteins that work as TFs has been more difficult. Here, we review numerous efforts to develop and explore pharmacologic inhibitors targeting oncogenic fusion transcription regulators and/or relevant TFs.

### 4.1. PML–RARα

PML–RARα is a transcription factor which underlies the pathogenesis of acute promyelocytic leukemia (APL). With the discovery of ATRA that, in pharmacological doses, selectively binds to the mutant TF to induce leukemia cell differentiation [157], APL has evolved from being the most malignant form of acute leukemia to a disease with excellent long-term survival rates. Development of non-chemotherapeutic drugs for acute leukemia has numerous advantages including relatively modest side-effects.

A balanced translocation t(15;17)(q24;q21) fuses the promyelocytic leukemia *PML* gene on chromosome 15 to the retinoic acid receptor alpha *RARA* gene on chromosome 17, and the resulting PML–RARα fusion protein is the master driver of APL [158]. PML has constitutive or transient interactions with more than 170 proteins with which it can be organized in subnuclear structures termed nuclear bodies (PML NBs) that regulate apoptosis, self-renewal of stem cells, senescence, and metabolism. The RARα protein is a member of the nuclear receptor superfamily that serves as a nuclear TF when activated by its cognate retinoid ligands. RARα acts as a differentiating agent of normal myeloid hematopoietic cells.Its activity is dependent on the presence of the ligand [159,160].

PML–RARα is a diagnostic hallmark of APL, the unique subtype of leukemias, which accounts for 10–15% of AML. PML–RARα behaves as an altered TF repressing RARα targets and antagonizes the formation and function of PML NBs [159]. The chimeric TF undergoes aberrant dimerization and assembly with corepressors to dysregulate normal RARα function, leading to suppression of different genes. This results in the arrest of granulocyte differentiation and malignant transformation of hematopoietic cells, given that cells are insensitive to physiological amounts of retinoid [160]. Treatment with high doses of ATRA, a ligand of RARα, in combination with chemotherapy could help to overcome the differentiation block leading to terminal differentiation of tumor cells and complete recovery in >90% cases [161]. At pharmacological doses, ATRA switches PML–RARα from a transcriptional repressor into an activator by inducing the release of corepressors and recruitment of coactivators [160,162]. ATRA interacts with the RARα portion of the fusion protein, thereby changing its configuration; this event induces degradation via the ubiquitin/proteasome system (UPS) [163]. Therefore, ATRA triggers rapid differentiation of leukemic cells into granulocytes, which correlates with remission in APL patients. However, with single-agent ATRA therapy, recoveries are usually transient, suggesting that differentiation alone cannot abolish self-renewal of tumor cells. Thus, ATRA combined with chemotherapy is a standard for treatment of high-risk patients.

Another potent anti-APL agent suitable both for relapsed patients, as well as for primary APL therapy, is arsenic trioxide (ATO) [164]. ATO is considerably more efficient than ATRA as a single agent and leads to degradation of the fusion protein by binding to the PML portion following SUMOylation. PML SUMOylation recruits RING finger protein 4 (RNF4) to PML NBs. RNF4 is a SUMO-dependent ubiquitin ligase that polyubiquitylates the PML moiety and targets the fused protein to the proteasome [164]. Various strategies of using ATO in APL treatment have been developed [165]. The initial results of the APL0406 trial (NCT00482833) showed that the combination of ATRA and ATO is at least not inferior to standard ATRA+chemotherapy in first-line therapy of low- or intermediate-risk APL [166]. Moreover, recent studies (APL2012 trial, NCT01987297) have demonstrated that the ATRA–ATO combination in both chemotherapy-replacing and -reducing settings in consolidation is effective as a traditional ATRA–chemo combination [17].

### 4.2. RUNX1–RUNX1T1

*RUNX1–RUNX1T1* (also known as *AML1–ETO*) is one of the most common chromosomal alterations found in AML. Perturbation of RUNX1–RUNX1T1 levels and its DNA binding affects chromatin accessibility and TF occupation at multiple gene loci associated with changes in gene expression levels [167]. *RUNX1–RUNX1T1*-mediated transcription program is a complex regulatory network promoting leukemic self-renewal and propagation. The *RUNX1–RUNX1T1* fusion has been detected in up to 5% of AML cases [168,169]. AML cells carrying t(8;21) have a morphologically distinct phenotype. This imparts a favorable prognosis in adults but a poor prognosis in children [170].

The *RUNX1* (also known as *AML1* or *CBFA2*) gene on chromosome 21 and the *RUNX1T1* (*ETO* or *MTG8*) gene on chromosome 8 join together in the course of t(8;21)(q22;q22) translocation. The resultant RUNX1–RUNX1T1 fusion protein is a leukemia-initiating TF that interferes with the hematopoietic master regulator RUNX1 function. RUNX1 is a key TF that, together with its heterodimerization partner, the core-binding factor beta (CBFβ), interacts with other TFs and transcriptional coregulators (the abovementioned PU.1, CEBPα, mSin3a, GATA1, etc.) to regulate genes involved in hematopoiesis [171]. These interactions are crucial for hematopoietic differentiation and myeloid development, and RUNX1 expression is transient and limited to erythropoiesis. The RUNX1–RUNX1T1 rearrangement leads to recruitment of RUNX1T1, whose level is low in normal hematopoietic cells but enhances due to t(8;21). RUNX1T1 recruits transcriptional repressors including *N*-Cor/SMRT, mSin3A, and HDAC that inhibit transcription of genes involved in hematopoiesis [169]. Essential for the oncogenic potential of RUNX1–RUNX1T1 is oligomerization of the chimeric fusion protein through the nervy homology region 2 (NHR2) within RUNX1T1 [172]. The resulting fusion product is a TF that regulates genes involved in stem and progenitor cell proliferation, differentiation, and function, ultimately blocking differentiation and AML development [173].

Although TFs are difficult to target, several promising attempts of RUNX–RUNX1T1 inhibition have been made. Current approaches to directly target RUNX1–RUNX1T1 in vitro include the use of small interfering (si) RNAs targeting the fusion site of chimeric mRNA or suppression of oligomerization by polypeptides or low-molecular-weight compounds [172,174,175,176,177]. Both methods work in AML cell cultures, achieving the loss of leukemia cell self-renewal and overcoming the block of myeloid differentiation. However, these approaches are difficult to apply in therapy due to poor pharmacokinetic properties and a complicated delivery. Nevertheless, application of targeting siRNA into anticancer therapy is intensively being investigated. Therapeutic targeting of AML fusion transcripts may be considered as a propitious approach of fusion inhibition.

Spirin and coworkers demonstrated the silencing of the *RUNX1–RUNX1T1* gene after short hairpin (sh) RNA coding vector transduction dramatically reduces the growth rate and leads to proapoptotic signaling in AML Kasumi-1 cells [178]. Furthermore, the authors performed transcriptional profiling of cells resistant to RUNX1–RUNX1T1 suppression and discovered upregulation of proliferative and prosurvival pathways in resistant cells. Thus, consistent evidence suggests that inhibition of RUNX1–RUNX1T1 (protein or gene) alone may be insufficient. Johnson and colleagues established the downregulation of the *RUNX1–RUNX1T1* oncogene expression in t(8;21) AML by microRNA let-7b targeting 3′-untranslated regions (UTRs) of fusion transcripts [179]. Remarkably, the chimeric gene *RUNX1–RUNX1T1* uses 3′UTRs of the *RUNX1T1* gene, which is not normally expressed in hematopoietic cells. The authors concluded that the mechanisms regulating *RUNX1–RUNX1T1* expression via 3′UTRs are therapeutically relevant.

Approaches to suppress RUNX1–RUNX1T1 include inhibition of interaction partners of the fusion protein. These interactions are critical for its subcellular localization and function. The development of inhibitors that prevent the formation of the RUNX1/CBFβ complex (CBFβ is a non-DNA-binding subunit that increases the affinity of the fusion protein for DNA and is critical for RUNX1–RUNX1T1 activity [180]) inspires confidence that this approach could be efficient [181]. The HDACs are an interaction partner of RUNX1–RUNX1T1 and could also be a target [108,182]. Indeed, HDAC inhibitors block leukemogenesis in RUNX1–RUNX1T1 cells [183]. Duque-Afonso and colleagues demonstrated that the HDAC 1 inhibitor entinostat relieves epigenetic silencing of genes mediated by RUNX1–RUNX1T1. In combination with decitabine, a DNA demethylating agent, entinostat decreases the viability and proliferation of AML cells that carry t(8;21) [184].

### 4.3. MLL

The *MLL* (*MLL1* renamed as lysine-specific methyltransferase 2A or *KMT2A*) gene on chromosome 11q23 is frequently disrupted by chromosomal rearrangements that occur in the unique group of acute leukemias. *MLL*-r including the translocations involving 11q23 with >30 sites resulting in *MLL* fusion genes have been described in ALL and in 5–10% of AML [185]. The *MLL* gene is affected by chromosomal translocations that fuse it in-frame to one of over 70 gene partners. The fused MLL acts as a TF and aberrantly regulates gene transcription while retaining H3K79 methyltransferase activity [186,187,188]. The best characterized function of MLL fusion proteins is maintenance of expression of *HOX* clusters [189], whose dysregulation leads to leukemic transformation [190,191]. Two regions in the MLL fusion are essential for its ability to induce leukemogenesis [186]. One is an *N*-terminal motif that binds with the transcription coactivators Menin [192,193] and PAF1C [194]. These interactions have been shown to recruit MLL fusion proteins to their target genes. The second region is the CXXC domain that binds specifically to nonmethylated CpG motifs in the genome [195]. The most common MLL fusion partners AF4, AF9, and ENL (nuclear proteins that act as transcription activators) include transcriptional activation domains important for tumorigenesis [196]. Thus, *MLL*-r forms the chimeric fusion oncoprotein (*MLL* and the partner) that regulates the expression of a set of critically important genes. Therefore, disrupting protein–protein interactions sounds intriguing for treatment of MLL-positive leukemias.

Menin (encoded by the *MEN1* gene) is an essential oncogenic cofactor for MLL oncoproteins in leukemogenesis. Association of Menin with MLL upregulates *HOXA9* or *MEIS1* that are critical for enhanced self-renewal in AML [193]. Moreover, Menin–MLL interaction in the context of the mutated nucleophosmin 1 (*NPM1*) gene is important for gene expression in AML [197].

The group of Grembecka and Cierpicki have developed and optimized a novel class of inhibitors of Menin–MLL fusion protein interaction [79,198,199,200,201,202]. Compounds MI-503, MI-538, MI-1481, and MI-3454 effectively suppressed the growth of leukemic cells with *MLL*-r in vitro and in vivo and did not affect the growth of leukemia without MLL mutation and normal hematopoiesis. Inhibition of the complex resulted in suppression of gene expression programs mediated by MLL fusion, such as the expression of *HOXA9* and *MEIS1* genes. A structurally close analogue of MI-3454 with similar antileukemic activity, KO-539, and another drug that aims to disrupt the Menin–MLL interactions, SNDX-5613, have been tested in clinical trials (NCT04067336, NCT04065399) [203]. Moreover, co-inhibition of HDAC and the Menin–MLL interaction displayed a highly synergistic antitumor activity in vitro and in vivo [18]. This finding provides a preclinical basis for further investigation of targeted strategy combining HDAC and Menin–MLL antagonists.

### 4.4. CBFβ–MYH11

In 1993, Liu and colleagues identified that the recurrent balanced chromosomal rearrangement inv(16)(p13q22) and its variant t(16;16)(p13;q22) in AML lead to fusion of genes coding for the transcriptional coactivator core-binding factor β *CBFB* and smooth muscle myosin heavy chain *MYH11,* resulting in the formation of *CBFB–MYH11* chimera [204]. The fusion protein CBFβ–MYH11 acts as a dominant repressor of CBFβ function, interacting with RUNX1 [205,206]. Thus, the CBFβ–MYH11 fusion plays an essential role in deregulation of genes involved in maintenance of a stem-cell phenotype and normal hematopoiesis [203].

Castailla and colleagues have demonstrated that, in a knock-in mouse model, the CBFβ–MYH11 fusion protein blocks a definitive hematopoiesis repressing the RUNX1/CBFβ function. A similar phenotype is observed in mice with the complete knockout of *runx1^−/−^* or *cbfb*^−/−^ [207,208,209]. The subsequent studies of this group demonstrated that formation of the CBFβ–MYH11 fusion is necessary but insufficient for leukemogenesis. Cooperation with additional genetic alterations (e.g., mutations in the *KIT*, *FLT3*, *NRAS*, and *KRAS* genes) is required for acute leukemia development [210,211,212,213].

The first anti-CBFβ–MYH11 inhibitor, AI-10-49, was reported in 2015 [214]. Its mechanism of action involves disruption of the protein–protein interaction between the fusion protein and RUNX1 via binding to the CBFβ portion of the chimera. Upon inhibition of CBFβ–MYH11/RUNX1 interaction by AI-10-49, RUNX1 represses the *MYC* expression by replacing the BAF complex component BRG1 with the Polycomb repressive complex component RING1B, thereby leading to apoptosis [215]. Combinations of AI-10-49 with JQ1 (see Section 2.2) revealed a strong synergy in inv(16) AML cells and a significant delay in leukemia development in mice. This compound is under development as an antileukemic drug and has been licensed by Systems Oncology, LLC for clinical development [203].

Richter and colleagues demonstrated that HDAC1 is a cofactor of CBFβ–MYH11 that regulates its activity. The HDAC1 inhibitor entinostat blocks the growth of CBFβ–MYH11-positive leukemia cells and promotes their differentiation, indicating that HDAC inhibitors may be useful for treatment of inv(16) AML [216].

The formation of CBFβ–MYH11 fusion occurs persistently in AML and is a highly specific hallmark of the disease. Recent studies indicate that the CBFβ–MYH11 fusion neoantigen is present on AML blasts, thereby enabling T-cell recognition and killing of tumor cells [217].

### 4.5. BCR–ABL1

BCR–ABL1 is a constitutively active tyrosine kinase formed as a result of chromosomal rearrangement t(9;22)(q34;q11). The formation of the BCR–ABL1 chimera is a critical event in CML pathogenesis. The rearrangement t(9;22)(q34;q11) results in the formation of an aberrant Philadelphia (Ph) chromosome. Molecular characterization revealed the occurrence of a new chimeric oncogene that consists of fused *BCR* and *ABL1* genes; the 5′ part of the *BCR* gene at 22q11 and the 3′ part of the *ABL1* tyrosine kinase-encoding gene at 9q34 are joined together, leading to the creation of a hybrid BCR–ABL1 oncoprotein with increased tyrosine kinase activity (reviewed in [218]). This is a classical example of a translocation common for all types of CML, some types of ALL, and AML. BCR–ABL1 is an almost perfect chemotherapeutic target. Approval of the selective inhibitor of BCR–ABL1 kinase activity, imatinib mesylate (Gleevec, Glivec, STI571), has commemorated the beginning of a new era of anticancer targeted medicine in the treatment of BCR–ABL1-positive leukemias [156]. However, the recognition that some patients experience relapse due to resistance-conferring point mutations within *BCR–ABL1* led to the development of second- and third-generation inhibitors [219]. Another approach in treatment of standard therapy-resistant Ph-positive leukemia is targeting signaling pathways governed by BCR–ABL1 fusion [220]. Asciminib, an allosteric inhibitor designed to specifically target the ABL myristoyl pocket (STAMP), demonstrated efficacy in situations of failure of other tyrosine kinase blockers, as well as in patients with compromised renal or liver functions [221,222,223,224]. Thus, asciminibcan potentially become an efficient substitute for early generations of BCR–ABL1 antagonists.

BCR–ABL1 activates a variety of pathways such as MAPK, JAK/STAT5, and PI3K/AKT. Autophosphorylation of BCR–ABL1 at Tyr177 promotes the formation of a GRB2 (growth factor receptor-bound 2) complex with GAB2 (GRB2-associated binder) and son-of-sevenless (SOS), triggering activation of RAS GTPase and recruitment of PI3K and the SH2-containing protein tyrosine phosphatase SHP2 [225,226,227]. Signaling downstream from RAS activates a MAPK (mitogen-activated protein kinase) pathway that supports cell proliferation. PI3K activates the serine/threonine kinase Akt which promotes survival by suppressing the activity of forkhead O (FOXO) TF [228], as well as enhances cell proliferation via activation of a proteasomal degradation of p27 through upregulation of Skp2, the protein of the SCF^Skp2^ E3 ubiquitin ligase [229], and activation of mTOR [230]. The signal transducer and activator of transcription STAT5 is activated through direct phosphorylation by BCR–ABL1 or indirectly through phosphorylation by JAK2 or Hck [231,232]. STAT5, but not JAK2, is extremely important for the maintenance of BCR–ABL1-positive leukemia [233]. Altogether, these signaling pathways modulate gene transcription.

STAT5 has a pivotal role in the resistance of leukemic cells to treatment with tyrosine kinase inhibitors and promotes survival of LSCs. Thus, blocking STAT5-mediated transcriptional activity is an important potentially druggable target [234]. In addition to phosphorylation on tyrosine by BCR–ABL1, the transcriptional activity of STAT5 requires dimerization via SH2 domains. Page and colleagues demonstrated that inhibition of STAT5 by selective compounds BP-1-075 and BP-1-108 targeting the SH2 domains has a potent antileukemic effect. The lead agent BP-1-108 showed negligible cytotoxicity in the bone marrow cells not expressing activated STAT5 [235]. Another approach to STAT5 inhibition involves interference with its nuclear translocation. Pimozide has been identified as a potential STAT5 inhibitor in BCR–ABL1-positive cells and in an AML mouse model [236]; however, this drug is efficacious at high concentrations and is not potent enough to be considered for clinical application [234].

Unfortunately, fused TFs in AML remain a difficult target for development of drug candidates. The abovementioned approaches including inhibition of protein–protein interactions are partially successful preclinically but less often clinically (Table 4). One of the most promising approaches that recently emerged for hematologic malignancies is PROTACs (proteolysis-targeting chimeras) [237]. PROTACs are artificial heterobifunctional small molecules that utilize the ubiquitin proteasome system to degrade the proteins of interest. The first part of the PROTAC is designed to selectively bind targeted protein, while the second part recruits E3 ubiquitin ligase. Because of their ability to target mutant or undruggable proteins including fusion proteins, PROTACs can be therapeutically advantageous compared to enzymatic inhibitors. The efficacy of this technology has been reported for inhibition of numerous oncoproteins in a variety of tumors including blood malignancies (reviewed in [237]). The safety, selectivity, and therapeutic efficacy of PROTACs in hematologic malignancies emerge as hot problems.

## 5. Drug Resistance and Transcriptional Modulators in Leukemia: Focus on Stemness

As a result of the successful development of different cytotoxic and target drugs, the mean 5year survival in leukemia surpassed 80% in recent decades [238]. Nevertheless, there are certain cohorts with poor prognosis; patients > 60 years with AML demonstrate only 40–60% of remission with overall 24% 5 year survival [239], while ALL is the leading cause of cancer related deaths among children [240]. Therapy failure is often associated with drug resistance; in addition to 35–45% of resistant tumors among newly diagnosed cases, relapsed tumors almost never respond to the previously used therapy [241]. Sometimes the genetic basis of this resistance cannot be found (reviewed in [4]); hematological malignancies (with the high rate of pediatric cases), especially AML, carry relatively low mutation burden. In AML, about 40% of resistant tumors show no signs of nonsynonymous coding mutations responsible for resistance [241].

Two main scenarios of nongenetic resistance emergence have been proposed [3]. The first scenario implies transcriptional plasticity [68,242]. This means that alterations in transcription of certain genes are mechanistically attributable to this type of resistance; importantly, this unfavorable phenotype can be prevented by drugs reducing transcriptional plasticity or overcome by epigenetic modulators [68,242,243,244].

Guo and colleagues analyzed the role of the *PVT1* enhancers in AML resistance to BRD4 inhibitors. *PVT1* is a noncoding RNA locus that normally acts as a tumor-suppressive chromatin boundary element, but it contains intergenic enhancers capable of driving *MYC* expression. *PVT1* enhancer-driven *MYC* expression was shown in murine and human cells resistant to BRD4 inactivation. Administration of the CDK7 inhibitor in combination with the BRD4 inhibitor interrupted RNA Pol II loading at the *PVT1–MYC* transcription complex, suppressing the growth of resistant cells [242]. Knoechel and colleagues, investigating therapeutic effects of γ-secretase inhibitors of NOTCH1 activation against T-ALL, noticed that resistance to these inhibitors was related to epigenetic modifications. In their study, BRD4 was found to occupy *MYC* and *BCL2* enhancers of resistant cells; BRD4 inhibitors successfully alleviated this resistant phenotype [243].

Another possible scenario is attributed to a small population of stem-like tumor cells with distinct transcriptional patterns, which are present at the diagnosis and are not eradicated by conventional therapy [2,68,243,244,245,246]. Stem-like states and epigenetic activation of driver oncogenes such as *MYC* are largely dependent on SE genes that are prone to an epigenetic switch because of their ‘all-or-nothing’ expression profile. Activation of lineage-specific genes can also help to overcome the resistance in the stem-like cells.

Shlush and colleagues proposed two similar non-genetic situations: a relapse from very few cells with hematopoietic stem phenotype and a relapse from a larger number of committed cells but with strong stem-like transcription patterns [245]. Resistance to BET inhibitors was associated with a stem-like Gr11^−^/CD11b1^−^phenotype. In the absence of the promoter-bound BRD4 (after its pharmacological inhibition), the transcription of key genes was governed by β-catenin. Genetic inactivation and pharmacologic inhibition of Wnt/β-catenin promoted a Gr11^+^/CD11b1^+^differentiated phenotype and restored sensitivity to I-BET [2]. Bell and colleagues modeled the whole process by establishing a cell line resistant to BET inhibitors; a nongenetic mechanism of this resistance was demonstrated. Lastly, inhibition of LSD1 leads to the formation of new enhancers densely occupied by BRD4 and leukemic stem-cell differentiation, which, despite not killing the cells, re-sensitized them to the BET inhibitor [68]. These examples bear evidence that transcription targeting drugs can successfully fill the niche at the second defense line against refractory/relapsed malignancies.

## 6. Conclusions

Along with the disclosure of specific mechanisms of gene regulation in tumor cells, as well as with the development of chemical and genetic tools to target these mechanisms, gene transcription became an important area of experimental and clinical investigations. In this respect, hematological malignancies represent a leading disorder where targeting transcription, as a monotherapy and/or in combinations with conventional chemotherapeutic and targeted drugs, emerges as a novel promising approach.

Of special interest are the attempts to combat blood cell plasticity (interpretable as a basis for tumor cell escape, emergence of drug resistance, and disease progression) via interference with transcriptional reprogramming. This unique mechanism, being commonly nonfunctional in the adult organism, becomes critical when cell survival or a switch in function requires rapid adaptation of the transcriptional machinery. Importantly, modern inhibitors of transcriptional reprogramming are virtually nontoxic over the course of a prolonged oral administration. These agents are attractive mainly for therapeutic combinations although, in particular situations (e.g., AML and other SE-associated tumors), inhibition of transcriptional reprogramming kinases becomes vital. Furthermore, experimental evidence is growing in support of the role of this mechanism in de novo acquisition of antileukemia drug resistance. These observations set the stage for combination strategies aimed at improving patient outcome. It remains to be elucidated whether the effects of patented pharmacological agents (including those in clinical trials) and investigational PROTAC degraders are similar, given that transcriptional kinases have both enzymatic enzyme-independent functions.

These considerations are likely to be attributable to other classes of transcription-regulating agents analyzed in the present review. Definitely, the relevance of individual transcriptional mechanisms to the biology of various blood tumors differs; therefore, general interventions could be of limited success. The field of transcriptional drugs is in its infancy. Nevertheless, the fundamental importance of this regulation and the perspective of initial studies provide a strong hope for patients and clinicians.

## Figures and Tables

**Table 1:** Functional roles of histone 3 modifications at lysine residues 4, 9, 27, and 79.

Type of Modification	H3K4	H3K9	H3K27	H3K79
Monomethylation (me1)	Activation (enhancers)	Activation	Activation	
Dimethylation (me2)	Activation (enhancers/promoters)	Repression	Repression	Activation
Trimethylation (me3)	Activation (promoters)	Repression	Repression (promoters)	Repression
Acetylation	Activation	Activation (enhancers/promoters)	Activation (enhancers)	Activation

**Table 2:** List of inhibitors of key chromatin regulators, their predicted mechanism of antitumor effects, and the stage of development.

Function	Targets	Inhibitors	Possible Mechanism	Clinical	Target Cells	Reference
Histone deacetylase						
	HDAC	Vorinostat		Phase II (NCT00097929)	AML, ALL, MML, B-cell lymphoma	[24]
		Panobinostat		Phase II (NCT01083602)		
		Belinostat		Phase I (NCT01273155)		
		Mocetinostat	Induction of TNF-⍺, NF-κB, and JAK/STATpathway	Phase II (NCT00324220)		[38]
		Entinostat	Inhibition of Bcl-2 and Mcl-1	Preclinical		[24]
		Romidepsin	Induction of c-Myc, HSP90, and p53	Phase II (NCT00106431, NCT00426764)		[39]
Histone acetyltransferase						
	BET	JQ1	Inhibition of Myc- dependent transcription, induction of HEXIM1	Preclinical	AML, ALL, MML, B-cell lymphoma	[54]
		OTX015		Phase I (NCT01713582)		[51]
		I-BET151		Preclinical		[56]
		I-BET762		Phase II (NCT01943851)		
		BI 894999		Preclinical		[50]
Histone demethylase						
H3K4, H3K9	LSD1	ORY-1001		Phase I (EudraCTNumber:2013-002447-29)	AML	[72]
		GSK2879552		Phase I (NCT02177812)		[73]
		IMG-7289	Activation of p53 and PUMA	Phase I/II (NCT03136185, NCT04254978, NCT04262141, NCT03136185, NCT04081220)	Myelofibrosis, thrombocythemia	[75]
		SP-2509	Activation of p53 and C/EBPα	Preclinical	AML	[76]
		NCD25	Induction of C/EBPα, PU.1 and RUNX1	Preclinical		[61]
		NCD38		Preclinical		
		Epiberberine		Preclinical		[70]
Histone methyltransferases						
H3K4	MLL1-complex	MM-401	Inhibition of *HOXA9* and/or *MEIS1*	Preclinical	*MLL*-r AML	[78]
H3K9	G9A	BIX-01294	Inhibition of HOXA9	Preclinical	HOXA9-overexpressed AML	[82]
		DCG066		Preclinical		[84]
		A-366		Preclinical		[83]
H3K27	EZH1/2	DZNep		Preclinical	*MLL*-r and other AML, B-cell lymphoma	[92]
		EPZ005687		Preclinical		[93]
		GSK126		Preclinical		[89]
		EI1		Preclinical		[94]
		Tazemetostat		Phase I/II (NCT01897571)		[95]
		UNC1999		Preclinical		[84]
		SAH-EZH2		Preclinical		[88]
H3K79	DOT1L	EPZ4777	Inhibition of *HOXA9* and/or *MEIS1*	Preclinical	*MLL*-r AML	[103]
		Pinometostat		Phase II (NCT03724084)		[105]
H4R3	PRMT1			Preclinical	*MLL*-r AML	[110]
Histoneacetyltransferase	KAT2A				AML, CML	[114]
Lysinacetiltransferase	CREBBP/EP300		*MYC*-transcription		AML, CML	[115]
Chromatin remodeling	BAF (BRD9 subunit)				AML	[121]

**Table 3:** List of transcriptional CDK inhibitors: predicted mechanism of antitumor efficacy and the stage of development.

Targets	Inhibitors	Possible Mechanism	Clinical	Target Cells	Reference
CDK7/9	Alvocidib	Inhibition of *MCL1*	Phase II (NCT03969420)	Relapsed/refractory AML	[132,133]
	Seliciclib		Preclinical		
	THZ1	Downregulation of SE-driven oncogene transcription	Preclinical	T-cell lymphoma, T-ALL	[136,137]
	CT7001		Preclinical	AML	[138,139]
	Dinaciclib	Inhibition of Mcl-1	Phase Ib (NCT03484520, NCT01650727)	Relapsed/refractory AML, relapsed/refractory CLL	
	JSH-009	Inhibition of *MCL1* and *MYC* genes and encoded proteins expression	Preclinical	AML	[134]
	Fadraciclib	Inhibition of *MCL1* and *BCL2*	Phase I (NCT04017546)	Relapsed/Refractory AML or MDS	[132]
	LDC067	Inhibition of *MYC* and *MCL1*	Preclinical	AML	[122]
CDK8/19	Cortistatin A	Inhibition of pSTAT1 S727, deregulation of SE-driven genes	Preclinical	AML and MLL	[47,145,146]
	Senexin A/B	Inhibition of pSTAT1 S727 and pSTAT5 S726	Preclinical	AML	[149]
	SEL120-34A	Inhibition of *MCL1*	Phase Ib (NCT04021368)	AML, High risk MDS	[140]
CDK12/13	THZ531	Downregulation DNA damage-related and SE-driven genes	Preclinical	Jurkat T-cell ALL	[153]

**Table 4:** Inhibitors of fusion TFs and their partners: predicted mechanism of action and the stage of development.

Function	Target	Inhibition Approach	Possible Mechanism	Clinical Trial Status	Target Cells	References
Transcription factor						
PML–RARα	RARα moiety	ATRA	Switching cofactors and induction of degradation via the UPS	Phase IV (NCT01987297) ATRA + chemotharapy/ATO	APL	[17]
	PML moiety	ATO	SUMOylation-mediated activation of UPS for fusion protein degradation	Phase IV (NCT01987297) ATO + ATRA	APL	[17]
RUNX1–RUNX1T1	Fusion mRNA	RNA interference	siRNA-mediated targeting of chimeric mRNA fusion site and subsequent silencing of fusion gene	Preclinical	AML cell lines Kasumi-1 and SKNO-1	[174]
	Fusion mRNA	RNA interference	shRNA-mediated targeting of chimeric mRNA fusion site and subsequent reduction of fusion gene expression	Preclinical	Kasumi-1	[178]
	Fusion mRNA	RNA interference	Post-transcriptional regulation via microRNA let-7b targeting the 3’UTR fusion transcript	Preclinical	t(8;21) AML patients samples, Kasumi-1 and SKNO-1	[179]
	Fusion protein	Introduction of a recombinant fusion polypeptide	Peptide-mediated suppression of oligomerization	Preclinical	Kasumi-1	[177]
	Fusion protein	Small-molecule inhibitor	Inhibition of protein–protein interaction	Preclinical	SKNO-1	[175]
	Fusion protein	Small-molecule inhibitor	Inhibition of tetramerization by blocking hot spots of oligomerization	Preclinical	Kasumi-1 and SKNO-1in vitro and in vivo	[177]
	Interaction partner HDAC	Small-molecule inhibitor entinostat(MS-275)	Relief of epigenetic silencing of genes mediated by oncofusion	Preclinical	Kasumi-1 and SKNO-1	[184]
MLL fusion oncoprotein	Interaction partner Menin	Small-molecule inhibitor (Menin–MLL inhibitor, MI)	Blockage of MLL–Menin interaction	Preclinical	*MLL*-r leukemia in vitro and in vivo	[79,198,199,200,201,202]
		Small-molecule inhibitor VTP50469	Displacement of Menin from protein complexes	Preclinical	*MLL*-r leukemia in vitro and in vivo	[80]
		Small-molecule inhibitor KO-539	Blockage of MLL–Menin interaction	Phase II (NCT04067336)	*MLL*-r leukemia	[202]
		Small-molecule inhibitor SNDX-5613		Phase I/II (NCT04065399)	*MLL*-r leukemia	[203]
CBFβ–MYH11	Interaction partner RUNX1	Small-molecule inhibitor AI-10-49	Binding to the CBFβ portion of the oncofusion and inhibiting the interaction with RUNX1	Preclinical	inv(16) AML patient samples, ME-1	[214]
	Interaction partner HDAC	Small-molecule inhibitor entinostat(MS-275)	Removal of epigenetic silencing of genes mediated by oncofusion	Preclinical	inv(16) AML in vitro and in vivo	[216]
Activation of signaling pathways						
BCR–ABL1	STAT5	Small-molecule inhibitors BP-1-075, BP-1-108	Suppression of oligomerization via SH2-domain binding	Preclinical	K562, MV-4-11	[235]
		Small-molecule inhibitor Pimozide	Decrease in STAT5 tyrosine phosphorylation and STAT5 target gene expression	Preclinical	CML patient samples, CML cell lines K562, KU812	[236]

## Abbreviations

ALL	acute lymphocytic leukemia
AML	acute myelogenous (myeloid) leukemia
APL	acute promyelocytic leukemia
ATO	arsenic trioxide
ATRA	all-*trans* retinoic acid
CDK	cyclin-dependent kinase
CML	chronic myelogenous (myeloid) leukemia
CTD	carboxy-terminal domain
HSC	hematopoietic stem cell
HAT	histone acetyltransferases
HDAC	histone deacetylase
HMT	histone methyltransferase
LSC	leukemia stem cell
MDS	myelodysplastic syndrome
MLL-r	*MLL* rearrangements
MPN	myeloproliferative neoplasm
NBs	nuclear bodies
Ph	Philadelphia chromosome
PROTACs	proteolysis-targeting chimeras
RNA Pol II	RNA polymerase II
SE	super-enhancer
TF	transcription factor
UPS	ubiquitin/proteasome system

## References

[1] Thoms J.A.I., Beck D., Pimanda J.E. (2019). Transcriptional networks in acute myeloid leukemia. Genes Chromosomes Cancer.

[2] Fong C.Y., Gilan O., Lam E.Y.N., Rubin A.F., Ftouni S., Tyler D., Stanley K., Sinha D., Yeh P., Morison J. (2015). BET inhibitor resistance emerges from leukaemia stem cells. Nature.

[3] Marine J.-C., Dawson S.-J., Dawson M.A. (2020). Non-genetic mechanisms of therapeutic resistance in cancer. Nat. Rev. Cancer.

[4] Bell C.C., Gilan O. (2020). Principles and mechanisms of non-genetic resistance in cancer. Br. J. Cancer.

[5] Glass J.L., Hassane D., Wouters B.J., Kunimoto H., Avellino R., Garrett-Bakelman F.E., Guryanova O.A., Bowman R., Redlich S., Intlekofer A.M. (2017). Epigenetic identity in AML depends on disruption of nonpromoter regulatory elements and is affected by antagonistic effects of mutations in epigenetic modifiers. Cancer Discov..

[6] Wang Y., Wu N., Liu D., Jin Y. (2017). Recurrent fusion genes in leukemia: An attractive target for diagnosis and treatment. Curr. Genom..

[7] Bruter A.V., Rodionova M.D., Varlamova E.A., Shtil A.A. (2021). Super-enhancers in the regulation of gene transcription: General aspects and antitumor targets. Acta Nat..

[8] Shi J., Whyte W.A., Zepeda-Mendoza C.J., Milazzo J.P., Shen C., Roe J.-S., Minder J.L., Mercan F., Wang E., Eckersley-Maslin M.A. (2013). Role of SWI/SNF in acute leukemia maintenance and enhancer-mediated Myc regulation. Genes Dev..

[9] Bahr C., von Paleske L., Uslu V.V., Remeseiro S., Takayama N., Ng S.W., Murison A., Langenfeld K., Petretich M., Scognamiglio R. (2018). A Myc enhancer cluster regulates normal and leukaemic hematopoietic stem cell hierarchies. Nature.

[10] He Y., Long W., Liu Q. (2019). Targeting super-enhancers as a therapeutic strategy for cancer treatment. Front. Pharmacol..

[11] Diesch J., Zwick A., Garz A.-K., Palau A., Buschbeck M., Götze K.S. (2016). A clinical-molecular update on azanucleoside-based therapy for the treatment of hematologic cancers. Clin. Epigenetics.

[12] Takei H., Kobayashi S.S. (2019). Targeting transcription factors in acute myeloid leukemia. Int. J. Hematol..

[13] Baker A., Gregory G.P., Verbrugge I., Kats L., Hilton J.J., Vidacs E., Lee E.M., Lock R.B., Zuber J., Shortt J. (2016). The CDK9 inhibitor dinaciclib exerts potent apoptotic and antitumor effects in preclinical models of MLL-rearranged acute myeloid leukemia. Cancer Res..

[14] Gerlach D., Tontsch-Grunt U., Baum A., Popow J., Scharn D., Hofmann M.H., Engelhardt H., Kaya O., Beck J., Schweifer N. (2018). The novel BET bromodomain inhibitor BI 894999 represses super-enhancer-associated transcription and synergizes with CDK9 inhibition in AML. Oncogene.

[15] McDermott M.S.J., Chumanevich A.A., Lim C.-U., Liang J., Chen M., Altilia S., Oliver D., Rae J.M., Shtutman M., Kiaris H. (2017). Inhibition of CDK8 mediator kinase suppresses estrogen dependent transcription and the growth of estrogen receptor positive breast cancer. Oncotarget.

[16] Mazan M., Majewska E., Mikula M., Wiklik K., Combik M., Golas A., Masiejczyk M., Fiedor E., Polak A., Cybulska M. (2019). SEL120, a potent and specific inhibitor of CDK8 induces complete remission in human patient derived xenograft models of acute myeloid leukemia. Proceedings of the Experimental and Molecular Therapeutics; American Association for Cancer Research.

[17] Chen L., Zhu H.-M., Li Y., Liu Q.-F., Hu Y., Zhou J.-F., Jin J., Hu J.-D., Liu T., Wu D.-P. (2021). Arsenic trioxide replacing or reducing chemotherapy in consolidation therapy for acute promyelocytic leukemia (APL2012 Trial). Proc. Natl. Acad. Sci. USA.

[18] Ye J., Zha J., Shi Y., Li Y., Yuan D., Chen Q., Lin F., Fang Z., Yu Y., Dai Y. (2019). Co-inhibition of HDAC and MLL-menin interaction targets MLL-rearranged acute myeloid leukemia cells via disruption of DNA damage checkpoint and DNA repair. Clin. Epigenetics.

[19] Park D.J., Kwon A., Cho B.-S., Kim H.-J., Hwang K.-A., Kim M., Kim Y. (2020). Characteristics of DNMT3a mutations in acute myeloid leukemia. Blood Res..

[20] Lauber C., Correia N., Trumpp A., Rieger M.A., Dolnik A., Bullinger L., Roeder I., Seifert M. (2020). Survival differences and associated molecular signatures of DNMT3A-mutant acute myeloid leukemia patients. Sci. Rep..

[21] Weissmann S., Alpermann T., Grossmann V., Kowarsch A., Nadarajah N., Eder C., Dicker F., Fasan A., Haferlach C., Haferlach T. (2012). Landscape of TET2 mutations in acute myeloid leukemia. Leukemia.

[22] Winters A.C., Bernt K.M. (2017). MLL-rearranged leukemias—An update on science and clinical approaches. Front. Pediatrics.

[23] Kim K.H., Roberts C.W.M. (2016). Targeting EZH2 in cancer. Nat. Med..

[24] San José-Enériz E., Gimenez-Camino N., Agirre X., Prosper F. (2019). HDAC inhibitors in acute myeloid leukemia. Cancers.

[25] Garcia-Carpizo V., Ruiz-Llorente S., Sarmentero J., Graña-Castro O., Pisano D.G., Barrero M.J. (2018). CREBBP/EP300 bromodomains are critical to sustain the GATA1/MYC regulatory axis in proliferation. Epigenetics Chromatin.

[26] Ghisi M., Johnstone R.W., Andreeff M. (2015). AML: Deacetylases. Targeted Therapy of Acute Myeloid Leukemia.

[27] Khan O., La Thangue N.B. (2012). HDAC inhibitors in cancer biology: Emerging mechanisms and clinical applications. Immunol. Cell Biol..

[28] Mottamal M., Zheng S., Huang T.L., Wang G. (2015). Histone deacetylase inhibitors in clinical studies as templates for new anticancer agents. Molecules.

[29] Peserico A., Simone C. (2011). Physical and functional HAT/HDAC interplay regulates protein acetylation balance. J. Biomed. Biotechnol..

[30] Cortiguera M.G., García-Gaipo L., Wagner S.D., León J., Batlle-López A., Delgado M.D. (2019). Suppression of BCL6 function by HDAC inhibitor mediated acetylation and chromatin modification enhances BET inhibitor effects in B-Cell lymphoma cells. Sci. Rep..

[31] Li A.G., Piluso L.G., Cai X., Gadd B.J., Ladurner A.G., Liu X. (2007). An acetylation switch in p53 mediates holo-TFIID recruitment. Mol. Cell.

[32] Bose P., Dai Y., Grant S. (2014). Histone deacetylase inhibitor (HDACI) mechanisms of action: Emerging insights. Pharmacol. Ther..

[33] Guo S.-Q., Zhang Y.-Z. (2012). Histone deacetylase inhibition: An important mechanism in the treatment of lymphoma. Cancer Biol. Med..

[34] Lu A., Luo H., Shi M., Wu G., Yuan Y., Liu J., Tang F. (2011). Design, synthesis and docking studies on benzamide derivatives as histone deacetylase inhibitors. Bioorg. Med. Chem. Lett..

[35] Patrick G.L., Patrick G.L. (2020). Chapter 16—Miscellaneous targets. Antimalarial Agents.

[36] Wagner J.M., Hackanson B., Lübbert M., Jung M. (2010). Histone deacetylase (HDAC) inhibitors in recent clinical trials for cancer therapy. Clin. Epigenetics.

[37] Lillico R., Lawrence C.K., Lakowski T.M. (2018). Selective DOT1L, LSD1, and HDAC class I inhibitors reduce HOXA9 expression in MLL-AF9 rearranged leukemia cells, but dysregulate the expression of many histone-modifying enzymes. J. Proteome Res..

[38] Boumber Y., Younes A., Garcia-Manero G. (2011). Mocetinostat (MGCD0103): A review of an isotype-specific histone deacetylase inhibitor. ExpertOpin. Investig. Drugs.

[39] Valdez B.C., Brammer J.E., Li Y., Murray D., Liu Y., Hosing C., Nieto Y., Champlin R.E., Andersson B.S. (2015). Romidepsin targets multiple survival signaling pathways in malignant T cells. Blood Cancer J..

[40] Piekarz R.L., Frye R., Prince H.M., Kirschbaum M.H., Zain J., Allen S.L., Jaffe E.S., Ling A., Turner M., Peer C.J. (2011). Phase 2 trial of romidepsin in patients with peripheral T-cell lymphoma. Blood.

[41] Garnier J.-M., Sharp P.P., Burns C.J. (2014). BET bromodomain inhibitors: A patent review. Expert Opin. Ther. Pat..

[42] Roe J.-S., Vakoc C.R. (2016). The essential transcriptional function of BRD4 in acute myeloid leukemia. Cold Spring Harb. Symp. Quant. Biol..

[43] Braun T., Gardin C. (2017). Investigational BET bromodomain protein inhibitors in early stage clinical trials for acute myelogenous leukemia (AML). Expert Opin. Investig. Drugs.

[44] Jiang G., Deng W., Liu Y., Wang C. (2020). General mechanism of JQ1 in inhibiting various types of cancer. Mol. Med. Rep..

[45] Alqahtani A., Choucair K., Ashraf M., Hammouda D.M., Alloghbi A., Khan T., Senzer N., Nemunaitis J. (2019). Bromodomain and extra-terminal motif inhibitors: A review of preclinical and clinical advances in cancer therapy. Future Sci. OA.

[46] Bechter O., Schöffski P. (2020). Make your best BET: The emerging role of BET inhibitor treatment in malignant tumors. Pharmacol. Ther..

[47] Pelish H.E., Liau B.B., Nitulescu I.I., Tangpeerachaikul A., Poss Z.C., Da Silva D.H., Caruso B.T., Arefolov A., Fadeyi O., Christie A.L. (2015). Mediator kinase inhibition further activates super-enhancer-associated genes in AML. Nature.

[48] Chaidos A., Caputo V., Karadimitris A. (2015). Inhibition of bromodomain and extra-terminal proteins (BET) as a potential therapeutic approach in hematological malignancies: Emerging preclinical and clinical evidence. Ther. Adv. Hematol..

[49] Rathert P., Roth M., Neumann T., Muerdter F., Roe J.-S., Muhar M., Deswal S., Cerny-Reiterer S., Peter B., Jude J. (2015). Transcriptional plasticity promotes primary and acquired resistance to BET inhibition. Nature.

[50] Tontsch-Grunt U., Rudolph D., Waizenegger I., Baum A., Gerlach D., Engelhardt H., Wurm M., Savarese F., Schweifer N., Kraut N. (2018). Synergistic activity of BET inhibitor BI 894999 with PLK inhibitor volasertib in AML in vitro and in vivo. Cancer Lett..

[51] Coudé M.-M., Braun T., Berrou J., Dupont M., Bertrand S., Masse A., Raffoux E., Itzykson R., Delord M., Riveiro M.E. (2015). BET inhibitor OTX015 targets BRD2 and BRD4 and decreases c-MYC in acute leukemia cells. Oncotarget.

[52] Delmore J.E., Issa G.C., Lemieux M.E., Rahl P.B., Shi J., Jacobs H.M., Kastritis E., Gilpatrick T., Paranal R.M., Qi J. (2011). BET bromodomain inhibition as a therapeutic strategy to target c-Myc. Cell.

[53] Chen C., Liu Y., Rappaport A.R., Kitzing T., Schultz N., Zhao Z., Shroff A.S., Dickins R.A., Vakoc C.R., Bradner J.E. (2014). MLL3 is a haploinsufficient 7q tumor suppressor in acute myeloid leukemia. Cancer Cell.

[54] Kang C., Kim C.-Y., Kim H.S., Park S.-P., Chung H.-M. (2018). The bromodomain inhibitor JQ1 enhances the responses to all-trans retinoic acid in HL-60 and MV4-11 leukemia cells. Int. J. Stem Cells.

[55] Gibbons H.R., Mi D.J., Farley V.M., Esmond T., Kaood M.B., Aune T.M. (2019). Bromodomain inhibitor JQ1 reversibly blocks IFN-γ production. Sci. Rep..

[56] Chaidos A., Caputo V., Gouvedenou K., Liu B., Marigo I., Chaudhry M.S., Rotolo A., Tough D.F., Smithers N.N., Bassil A.K. (2014). Potent antimyeloma activity of the novel bromodomain inhibitors I-BET151 and I-BET762. Blood.

[57] Stewart H.J.S., Horne G.A., Bastow S., Chevassut T.J.T. (2013). BRD4 Associates with p53 in DNMT3A-Mutated Leukemia Cells and Is Implicated in Apoptosis by the Bromodomain Inhibitor JQ1. Cancer Med..

[58] Maggisano V., Celano M., Malivindi R., Barone I., Cosco D., Mio C., Mignogna C., Panza S., Damante G., Fresta M. (2019). Nanoparticles loaded with the BET inhibitor JQ1 block the growth of triple negative breast cancer cells in vitro and in vivo. Cancers.

[59] Cusan M., Cai S.F., Mohammad H.P., Krivtsov A., Chramiec A., Loizou E., Witkin M.D., Smitheman K.N., Tenen D.G., Ye M. (2018). LSD1 inhibition exerts its antileukemic effect by recommissioning PU.1- and C/EBPα-dependent enhancers in AML. Blood.

[60] Magliulo D., Bernardi R., Messina S. (2018). Lysine-specific demethylase 1A as a promising target in acute myeloid leukemia. Front. Oncol..

[61] Sugino N., Kawahara M., Tatsumi G., Kanai A., Matsui H., Yamamoto R., Nagai Y., Fujii S., Shimazu Y., Hishizawa M. (2017). A novel LSD1 inhibitor NCD38 ameliorates MDS-related leukemia with complex karyotype by attenuating leukemia programs via activating super-enhancers. Leukemia.

[62] Yamamoto R., Kawahara M., Ito S., Satoh J., Tatsumi G., Hishizawa M., Suzuki T., Andoh A. (2018). Selective dissociation between LSD1 and GFI1B by a LSD1 inhibitor NCD38 induces the activation of ERG super-enhancer in erythroleukemia cells. Oncotarget.

[63] Fang Y., Liao G., Yu B. (2019). LSD1/KDM1A inhibitors in clinical trials: Advances and prospects. J. Hematol. Oncol..

[64] Gu F., Lin Y., Wang Z., Wu X., Ye Z., Wang Y., Lan H. (2020). Biological roles of LSD1 beyond its demethylase activity. Cell. Mol. Life Sci..

[65] Wingelhofer B., Somervaille T.C.P. (2019). Emerging epigenetic therapeutic targets in acute myeloid leukemia. Front. Oncol..

[66] Heintzman N.D., Stuart R.K., Hon G., Fu Y., Ching C.W., Hawkins R.D., Barrera L.O., Van Calcar S., Qu C., Ching K.A. (2007). Distinct and predictive chromatin signatures of transcriptional promoters and enhancers in the human genome. Nat. Genet..

[67] Kerenyi M.A., Shao Z., Hsu Y.-J., Guo G., Luc S., O’Brien K., Fujiwara Y., Peng C., Nguyen M., Orkin S.H. (2013). Histone demethylase Lsd1 represses hematopoietic stem and progenitor cell signatures during blood cell maturation. eLife.

[68] Bell C.C., Fennell K.A., Chan Y.-C., Rambow F., Yeung M.M., Vassiliadis D., Lara L., Yeh P., Martelotto L.G., Rogiers A. (2019). Targeting enhancer switching overcomes non-genetic drug resistance in acute myeloid leukaemia. Nat. Commun..

[69] Khan I., Eklund E.E., Gartel A.L. (2021). Therapeutic vulnerabilities of transcription factors in AML. Mol. Cancer Ther..

[70] Li Z.-R., Suo F.-Z., Guo Y.-J., Cheng H.-F., Niu S.-H., Shen D.-D., Zhao L.-J., Liu Z.-Z., Maa M., Yu B. (2020). Natural protoberberine alkaloids, identified as potent selective LSD1 inhibitors, induce AML cell differentiation. Bioorg. Chem..

[71] Bose P., Konopleva M.Y. (2018). ORY-1001: Overcoming the differentiation block in AML. Cancer Cell.

[72] Maes T., Mascaró C., Tirapu I., Estiarte A., Ciceri F., Lunardi S., Guibourt N., Perdones A., Lufino M.M.P., Somervaille T.C.P. (2018). ORY-1001, a potent and selective covalent KDM1A inhibitor, for the treatment of acute leukemia. Cancer Cell.

[73] Smitheman K.N., Severson T.M., Rajapurkar S.R., McCabe M.T., Karpinich N., Foley J., Pappalardi M.B., Hughes A., Halsey W., Thomas E. (2019). Lysine specific demethylase 1 inactivation enhances differentiation and promotes cytotoxic response when combined with all-trans retinoic acid in acute myeloid leukemia across subtypes. Hematologica.

[74] McLornan D., Percy M., McMullin M.F. (2006). JAK2 V617F: A single mutation in the myeloproliferative group of disorders. Ulster Med. J..

[75] Jutzi J.S., Kleppe M., Dias J., Staehle H.F., Shank K., Teruya-Feldstein J., Gambheer S.M.M., Dierks C., Rienhoff H.Y., Levine R.L. (2018). LSD1 inhibition prolongs survival in mouse models of MPN by selectively targeting the disease clone. Hemasphere.

[76] Fiskus W., Sharma S., Shah B., Portier B.P., Devaraj S.G.T., Liu K., Iyer S.P., Bearss D., Bhalla K.N. (2014). Highly effective combination of LSD1 (KDM1A) antagonist and pan-histone deacetylase inhibitor against human AML cells. Leukemia.

[77] Fang Y., Yang C., Yu Z., Li X., Mu Q., Liao G., Yu B. (2020). Natural products as LSD1 inhibitors for cancer therapy. Acta Pharm. Sin. B.

[78] Cao F., Townsend E.C., Karatas H., Xu J., Li L., Lee S., Liu L., Chen Y., Ouillette P., Zhu J. (2014). Targeting MLL1 H3K4 methyltransferase activity in mixed-lineage leukemia. Mol. Cell.

[79] He S., Senter T.J., Pollock J., Han C., Upadhyay S.K., Purohit T., Gogliotti R.D., Lindsley C.W., Cierpicki T., Stauffer S.R. (2014). High-affinity small-molecule inhibitors of the menin-mixed lineage leukemia (MLL) interaction closely mimic a natural protein-protein interaction. J. Med. Chem..

[80] Krivtsov A.V., Evans K., Gadrey J.Y., Eschle B.K., Hatton C., Uckelmann H.J., Ross K.N., Perner F., Olsen S.N., Pritchard T. (2019). A menin-MLL inhibitor induces specific chromatin changes and eradicates disease in models of MLL-rearranged leukemia. Cancer Cell.

[81] Ali A., Tyagi S. (2017). Diverse roles of WDR5-RbBP5-ASH2L-DPY30 (WRAD) complex in the functions of the SET1 histone methyltransferase family. J. Biosci..

[82] Jang J.E., Eom J.-I., Jeung H.-K., Chung H., Kim Y.R., Kim J.S., Cheong J.-W., Min Y.H. (2020). PERK/NRF2 and autophagy form a resistance mechanism against G9a inhibition in leukemia stem cells. J. Exp. Clin. Cancer Res..

[83] Pappano W.N., Guo J., He Y., Ferguson D., Jagadeeswaran S., Osterling D.J., Gao W., Spence J.K., Pliushchev M., Sweis R.F. (2015). The histone methyltransferase inhibitor A-366 uncovers a role for G9a/GLP in the epigenetics of leukemia. PLoS ONE.

[84] Kondengaden S.M., Luo L.-F., Huang K., Zhu M., Zang L., Bataba E., Wang R., Luo C., Wang B., Li K.K. (2016). Discovery of novel small molecule inhibitors of lysine methyltransferase G9a and their mechanism in leukemia cell lines. Eur. J. Med. Chem..

[85] Lehnertz B., Pabst C., Su L., Miller M., Liu F., Yi L., Zhang R., Krosl J., Yung E., Kirschner J. (2014). The methyltransferase G9a regulates HoxA9-dependent transcription in AML. Genes Dev..

[86] Cubillos-Ruiz J.R., Bettigole S.E., Glimcher L.H. (2017). Tumorigenic and immunosuppressive effects of endoplasmic reticulum stress in cancer. Cell.

[87] Xu B., On D.M., Ma A., Parton T., Konze K.D., Pattenden S.G., Allison D.F., Cai L., Rockowitz S., Liu S. (2015). Selective inhibition of EZH2 and EZH1 enzymatic activity by a small molecule suppresses MLL-rearranged leukemia. Blood.

[88] Kim W., Bird G.H., Neff T., Guo G., Kerenyi M.A., Walensky L.D., Orkin S.H. (2013). Targeted disruption of the EZH2-EED complex inhibits EZH2-dependent cancer. Nat. Chem. Biol..

[89] McCabe M.T., Ott H.M., Ganji G., Korenchuk S., Thompson C., Van Aller G.S., Liu Y., Graves A.P., Della Pietra A., Diaz E. (2012). EZH2 inhibition as a therapeutic strategy for lymphoma with EZH2-activating mutations. Nature.

[90] McCabe M.T., Graves A.P., Ganji G., Diaz E., Halsey W.S., Jiang Y., Smitheman K.N., Ott H.M., Pappalardi M.B., Allen K.E. (2012). Mutation of A677 in histone methyltransferase EZH2 in human B-cell lymphoma promotes hypertrimethylation of histone H3 on lysine 27 (H3K27). Proc. Natl. Acad. Sci. USA.

[91] Majer C.R., Jin L., Scott M.P., Knutson S.K., Kuntz K.W., Keilhack H., Smith J.J., Moyer M.P., Richon V.M., Copeland R.A. (2012). A687V EZH2 is a gain-of-function mutation found in lymphoma patients. FEBS Lett..

[92] Momparler R.L., Idaghdour Y., Marquez V.E., Momparler L.F. (2012). Synergistic antileukemic action of a combination of inhibitors of DNA methylation and histone methylation. Leuk. Res..

[93] Knutson S.K., Wigle T.J., Warholic N.M., Sneeringer C.J., Allain C.J., Klaus C.R., Sacks J.D., Raimondi A., Majer C.R., Song J. (2012). A selective inhibitor of EZH2 blocks H3K27 methylation and kills mutant lymphoma cells. Nat. Chem. Biol..

[94] Qi W., Chan H., Teng L., Li L., Chuai S., Zhang R., Zeng J., Li M., Fan H., Lin Y. (2012). Selective inhibition of Ezh2 by a small molecule inhibitor blocks tumor cells proliferation. Proc. Natl. Acad. Sci. USA.

[95] Knutson S.K., Warholic N.M., Wigle T.J., Klaus C.R., Allain C.J., Raimondi A., Porter Scott M., Chesworth R., Moyer M.P., Copeland R.A. (2013). Durable tumor regression in genetically altered malignant rhabdoid tumors by inhibition of methyltransferase EZH2. Proc. Natl. Acad. Sci. USA.

[96] Italiano A., Soria J.-C., Toulmonde M., Michot J.-M., Lucchesi C., Varga A., Coindre J.-M., Blakemore S.J., Clawson A., Suttle B. (2018). Tazemetostat, an EZH2 inhibitor, in relapsed or refractory B-cell non-Hodgkin lymphoma and advanced solid tumours: A first-in-human, open-label, phase 1 study. Lancet Oncol..

[97] Tsai C.T., So C.W.E. (2017). Epigenetic therapies by targeting aberrant histone methylome in AML: Molecular mechanisms, current preclinical and clinical development. Oncogene.

[98] Deshpande A.J., Deshpande A., Sinha A.U., Chen L., Chang J., Cihan A., Fazio M., Chen C.-W., Zhu N., Koche R. (2014). AF10 regulates progressive H3K79 methylation and HOX gene expression in diverse AML subtypes. Cancer Cell.

[99] Campbell C.T., Haladyna J.N., Drubin D.A., Thomson T.M., Maria M.J., Yamauchi T., Waters N.J., Olhava E.J., Pollock R.M., Smith J.J. (2017). Mechanisms of pinometostat (EPZ-5676) treatment-emergent resistance in MLL-rearranged leukemia. Mol. Cancer Ther..

[100] Kim W., Choi M., Kim J.-E. (2014). The histone methyltransferase Dot1/DOT1L as a critical regulator of the cell cycle. Cell Cycle.

[101] Bernt K.M., Zhu N., Sinha A.U., Vempati S., Faber J., Krivtsov A.V., Feng Z., Punt N., Daigle A., Bullinger L. (2011). MLL-rearranged leukemia is dependent on aberrant H3K79 methylation by DOT1L. Cancer Cell.

[102] Grigsby S.M., Friedman A., Chase J., Waas B., Ropa J., Serio J., Shen C., Muntean A.G., Maillard I., Nikolovska-Coleska Z. (2021). Elucidating the importance of DOT1L recruitment in MLL-AF9 leukemia and hematopoiesis. Cancers.

[103] Daigle S.R., Olhava E.J., Therkelsen C.A., Majer C.R., Sneeringer C.J., Song J., Johnston L.D., Scott M.P., Smith J.J., Xiao Y. (2011). Selective killing of mixed lineage leukemia cells by a potent small-molecule DOT1L inhibitor. Cancer Cell.

[104] Stein E.M., Garcia-Manero G., Rizzieri D.A., Tibes R., Berdeja J.G., Savona M.R., Jongen-Lavrenic M., Altman J.K., Thomson B., Blakemore S.J. (2018). The DOT1L inhibitor pinometostat reduces H3K79 methylation and has modest clinical activity in adult acute leukemia. Blood.

[105] Daigle S.R., Olhava E.J., Therkelsen C.A., Basavapathruni A., Jin L., Boriack-Sjodin P.A., Allain C.J., Klaus C.R., Raimondi A., Scott M.P. (2013). Potent inhibition of DOT1L as treatment of MLL-fusion leukemia. Blood.

[106] Kaushik S., Liu F., Veazey K.J., Gao G., Das P., Neves L.F., Lin K., Zhong Y., Lu Y., Giuliani V. (2018). Genetic deletion or small-molecule inhibition of the arginine methyltransferase PRMT5 exhibit anti-tumoral activity in mouse models of MLL-rearranged AML. Leukemia.

[107] Jarrold J., Davies C.C. (2019). PRMTs and arginine methylation: Cancer’s best-kept secret?. Trends Mol. Med..

[108] van der Kouwe E., Staber P.B. (2019). RUNX1-ETO: Attacking the epigenome for genomic instable leukemia. Int. J. Mol. Sci..

[109] He X., Zhu Y., Lin Y.-C., Li M., Du J., Dong H., Sun J., Zhu L., Wang H., Ding Z. (2019). PRMT1-mediated FLT3 arginine methylation promotes maintenance of FLT3-ITD^+^ acute myeloid leukemia. Blood.

[110] Cheung N., Fung T.K., Zeisig B.B., Holmes K., Rane J.K., Mowen K.A., Finn M.G., Lenhard B., Chan L.C., So C.W.E. (2016). Targeting aberrant epigenetic networks mediated by PRMT1 and KDM4C in acute myeloid leukemia. Cancer Cell.

[111] Arede L., Pina C. (2021). Buffering noise: KAT2A modular contributions to stabilization of transcription and cell identity in cancer and development. Exp. Hematol..

[112] Xiao M.-F., Huang Y.-M., Lu Z., Lin Z.-P. (2020). Expression and mechanism of KAT2A and CDK4/CDK6 in children with acute leukemia. Zhongguo Shi Yan Xue Ye Xue Za Zhi.

[113] Arede L., Foerner E., Wind S., Kulkarni R., Domingues A.F., Kleinwaechter S., Gupta S., Scheer E., Tora L., Pina C. (2020). Unique roles of ATAC and SAGA-KAT2A complexes in normal and malignant hematopoiesis. bioRxiv.

[114] Tzelepis K., Koike-Yusa H., De Braekeleer E., Li Y., Metzakopian E., Dovey O.M., Mupo A., Grinkevich V., Li M., Mazan M. (2016). A CRISPR dropout screen identifies genetic vulnerabilities and therapeutic targets in acute myeloid leukemia. Cell Rep..

[115] Crawford T.D., Romero F.A., Lai K.W., Tsui V., Taylor A.M., de Leon Boenig G., Noland C.L., Murray J., Ly J., Choo E.F. (2016). Discovery of a potent and selective in vivo probe (GNE-272) for the bromodomains of CBP/EP300. J. Med. Chem..

[116] Alfert A., Moreno N., Kerl K. (2019). The BAF complex in development and disease. Epigenetics Chromatin.

[117] Hodges C., Kirkland J.G., Crabtree G.R. (2016). The many roles of BAF (mSWI/SNF) and PBAF complexes in cancer. Cold Spring Harb. Perspect. Med..

[118] Lu C., Allis C.D. (2017). SWI/SNF complex in cancer. Nat. Genet..

[119] Bakshi R., Hassan M.Q., Pratap J., Lian J.B., Montecino M.A., van Wijnen A.J., Stein J.L., Imbalzano A.N., Stein G.S. (2010). The human SWI/SNF complex associates with RUNX1 to control transcription of hematopoietic target genes. J. Cell. Physiol..

[120] Pottier N., Yang W., Assem M., Panetta J.C., Pei D., Paugh S.W., Cheng C., Den Boer M.L., Relling M.V., Pieters R. (2008). The SWI/SNF chromatin-remodeling complex and glucocorticoid resistance in acute lymphoblastic leukemia. J. Natl. Cancer Inst..

[121] Remillard D., Buckley D.L., Paulk J., Brien G.L., Sonnett M., Seo H.-S., Dastjerdi S., Wühr M., Dhe-Paganon S., Armstrong S.A. (2017). Degradation of the BAF complex factor BRD9 by heterobifunctional ligands. Angew. Chem. Int. Ed. Engl..

[122] Galbraith M.D., Bender H., Espinosa J.M. (2019). Therapeutic targeting of transcriptional cyclin-dependent kinases. Transcription.

[123] Chou J., Quigley D.A., Robinson T.M., Feng F.Y., Ashworth A. (2020). Transcription-associated cyclin-dependent kinases as targets and biomarkers for cancer therapy. Cancer Discov..

[124] Cai D., Latham V.M., Zhang X., Shapiro G.I. (2006). Combined depletion of cell cycle and transcriptional cyclin-dependent kinase activities induces apoptosis in cancer cells. Cancer Res..

[125] Tibes R., Bogenberger J.M. (2019). Transcriptional silencing of MCL-1 through cyclin-dependent kinase inhibition in acute myeloid leukemia. Front. Oncol..

[126] Philip S., Kumarasiri M., Teo T., Yu M., Wang S. (2018). Cyclin-dependent kinase 8: A new hope in targeted cancer therapy?. J. Med. Chem..

[127] Roninson I.B., Győrffy B., Mack Z.T., Shtil A.A., Shtutman M.S., Chen M., Broude E.V. (2019). Identifying cancers impacted by CDK8/19. Cells.

[128] Lee D.J., Zeidner J.F. (2019). Cyclin-dependent kinase (CDK) 9 and 4/6 inhibitors in acute myeloid leukemia (AML): A promising therapeutic approach. Expert Opin. Investig. Drugs.

[129] Sampathi S., Acharya P., Zhao Y., Wang J., Stengel K.R., Liu Q., Savona M.R., Hiebert S.W. (2019). The CDK7 inhibitor THZ1 alters RNA polymerase dynamics at the 5’ and 3’ ends of genes. Nucleic Acids Res..

[130] Ren Y., Brown V., Hu S., Lopez J., Miljovska S., Schmidt D., Bradley M., Sprott K., Olson E., Fritz C.C. (2015). Targeting transcriptional dependency in acute myeloid leukemia (AML) with a covalent inhibitor of transcriptional kinase CDK7. Blood.

[131] Franco L.C., Morales F., Boffo S., Giordano A. (2018). CDK9: A key player in cancer and other diseases. J. Cell. Biochem..

[132] Frame S., Saladino C., MacKay C., Atrash B., Sheldrake P., McDonald E., Clarke P.A., Workman P., Blake D., Zheleva D. (2020). Fadraciclib (CYC065), a novel CDK inhibitor, targets key pro-survival and oncogenic pathways in cancer. PLoS ONE.

[133] Kadia T.M., Kantarjian H.M., Konopleva M. (2019). Myeloid cell leukemia-1 dependence in acute myeloid leukemia: A novel approach to patient therapy. Oncotarget.

[134] Wang L., Hu C., Wang A., Chen C., Wu J., Jiang Z., Zou F., Yu K., Wu H., Liu J. (2020). Discovery of a novel and highly selective CDK9 kinase inhibitor (JSH-009) with potent antitumor efficacy in preclinical acute myeloid leukemia models. Investig. New Drugs.

[135] Kwiatkowski N., Zhang T., Rahl P.B., Abraham B.J., Reddy J., Ficarro S.B., Dastur A., Amzallag A., Ramaswamy S., Tesar B. (2014). Targeting transcription regulation in cancer with a covalent CDK7 inhibitor. Nature.

[136] Li B.-B., Wang B., Zhu C.-M., Tang D., Pang J., Zhao J., Sun C.-H., Qiu M.-J., Qian Z.-R. (2019). Cyclin-dependent kinase 7 inhibitor THZ1 in cancer therapy. Chronic Dis. Transl. Med..

[137] Abudureheman T., Xia J., Li M.-H., Zhou H., Zheng W.-W., Zhou N., Shi R.-Y., Zhu J.-M., Yang L.-T., Chen L. (2021). CDK7 inhibitor THZ1 induces the cell apoptosis of B-Cell acute lymphocytic leukemia by perturbing cellular metabolism. Front. Oncol..

[138] Clark K., Ainscow E., Peall A., Thomson S., Leishman A., Elaine S., Ali S., Coombes R., Barrett A., Bahl A.K. (2017). CT7001, a novel orally bio-available CDK7 inhibitor, is highly active in in-vitro and in-vivo models of AML. Blood.

[139] Patel H., Periyasamy M., Sava G.P., Bondke A., Slafer B.W., Kroll S.H.B., Barbazanges M., Starkey R., Ottaviani S., Harrod A. (2018). ICEC0942, an orally bioavailable selective inhibitor of CDK7 for cancer treatment. Mol. Cancer Ther..

[140] Rzymski T., Mikula M., Żyłkiewicz E., Dreas A., Wiklik K., Gołas A., Wójcik K., Masiejczyk M., Wróbel A., Dolata I. (2017). SEL120-34A is a novel CDK8 inhibitor active in AML cells with high levels of serine phosphorylation of STAT1 and STAT5 transactivation domains. Oncotarget.

[141] Zhao X., Feng D., Wang Q., Abdulla A., Xie X.-J., Zhou J., Sun Y., Yang E.S., Liu L.-P., Vaitheesvaran B. (2012). Regulation of lipogenesis by cyclin-dependent kinase 8-mediated control of SREBP-1. J. Clin. Investig..

[142] Zhao J., Ramos R., Demma M. (2013). CDK8 regulates E2F1 transcriptional activity through S375 phosphorylation. Oncogene.

[143] Poss Z.C., Ebmeier C.C., Odell A.T., Tangpeerachaikul A., Lee T., Pelish H.E., Shair M.D., Dowell R.D., Old W.M., Taatjes D.J. (2016). Identification of mediator kinase substrates in human cells using cortistatin A and quantitative phosphoproteomics. Cell Rep..

[144] Kapoor A., Goldberg M.S., Cumberland L.K., Ratnakumar K., Segura M.F., Emanuel P.O., Menendez S., Vardabasso C., Leroy G., Vidal C.I. (2010). The histone variant macroH2A suppresses melanoma progression through regulation of CDK8. Nature.

[145] Nitulescu I.I., Meyer S.C., Wen Q.J., Crispino J.D., Lemieux M.E., Levine R.L., Pelish H.E., Shair M.D. (2017). Mediator kinase phosphorylation of STAT1 S727 promotes growth of neoplasms with JAK-STAT activation. EBioMedicine.

[146] Solum E., Hansen T.V., Aesoy R., Herfindal L. (2020). New CDK8 inhibitors as potential anti-leukemic agents—Design, synthesis and biological evaluation. Bioorg. Med. Chem..

[147] Menzl I., Witalisz-Siepracka A., Sexl V. (2019). CDK8-novel therapeutic opportunities. Pharmaceuticals.

[148] Cee V.J., Chen D.Y.-K., Lee M.R., Nicolaou K.C. (2009). Cortistatin A is a high-affinity ligand of protein kinases ROCK, CDK8, and CDK11. Angew. Chem. Int. Ed. Engl..

[149] Porter D.C., Farmaki E., Altilia S., Schools G.P., West D.K., Chen M., Chang B.-D., Puzyrev A.T., Lim C.-U., Rokow-Kittell R. (2012). Cyclin-dependent kinase 8 mediates chemotherapy-induced tumor-promoting paracrine activities. Proc. Natl. Acad. Sci. USA.

[150] Menzl I., Zhang T., Berger-Becvar A., Grausenburger R., Heller G., Prchal-Murphy M., Edlinger L., Knab V.M., Uras I.Z., Grundschober E. (2019). A kinase-independent role for CDK8 in BCR-ABL1^+^ leukemia. Nat. Commun..

[151] Tadesse S., Duckett D.R., Monastyrskyi A. (2021). The promise and current status of CDK12/13 inhibition for the treatment of cancer. Future Med. Chem..

[152] Fan Z., Devlin J.R., Hogg S.J., Doyle M.A., Harrison P.F., Todorovski I., Cluse L.A., Knight D.A., Sandow J.J., Gregory G. (2020). CDK13 cooperates with CDK12 to control global RNA polymerase II processivity. Sci. Adv..

[153] Zhang T., Kwiatkowski N., Olson C.M., Dixon-Clarke S.E., Abraham B.J., Greifenberg A.K., Ficarro S.B., Elkins J.M., Liang Y., Hannett N.M. (2016). Covalent targeting of remote cysteine residues to develop CDK12 and CDK13 inhibitors. Nat. Chem. Biol..

[154] Gao Y., Zhang T., Terai H., Ficarro S.B., Kwiatkowski N., Hao M.-F., Sharma B., Christensen C.L., Chipumuro E., Wong K.-K. (2018). Overcoming resistance to the THZ series of covalent transcriptional CDK inhibitors. Cell Chem. Biol..

[155] Xu Y., Milazzo J.P., Somerville T.D.D., Tarumoto Y., Huang Y.-H., Ostrander E.L., Wilkinson J.E., Challen G.A., Vakoc C.R. (2018). A TFIID-SAGA perturbation that targets MYB and suppresses acute myeloid leukemia. Cancer Cell.

[156] Druker B.J., Talpaz M., Resta D.J., Peng B., Buchdunger E., Ford J.M., Lydon N.B., Kantarjian H., Capdeville R., Ohno-Jones S. (2001). Efficacy and safety of a specific inhibitor of the BCR-ABL tyrosine kinase in chronic myeloid leukemia. N. Engl. J. Med..

[157] Zhou G.-B., Zhang J., Wang Z.-Y., Chen S.-J., Chen Z. (2007). Treatment of acute promyelocytic leukaemia with all-trans retinoic acid and arsenic trioxide: A paradigm of synergistic molecular targeting therapy. Philos. Trans. R. Soc. Lond. B Biol. Sci..

[158] Zhao J., Liang J.-W., Xue H.-L., Shen S.-H., Chen J., Tang Y.-J., Yu L.-S., Liang H.-H., Gu L.-J., Tang J.-Y. (2019). The genetics and clinical characteristics of children morphologically diagnosed as acute promyelocytic leukemia. Leukemia.

[159] Hsu K.-S., Kao H.-Y. (2018). PML: Regulation and multifaceted function beyond tumor suppression. Cell Biosci..

[160] Liquori A., Ibañez M., Sargas C., Sanz M.Á., Barragán E., Cervera J. (2020). Acute promyelocytic leukemia: A constellation of molecular events around a single PML-RARA fusion gene. Cancers.

[161] Lo-Coco F., Avvisati G., Vignetti M., Thiede C., Orlando S.M., Iacobelli S., Ferrara F., Fazi P., Cicconi L., Di Bona E. (2013). Retinoic acid and arsenic trioxide for acute promyelocytic leukemia. N. Engl. J. Med..

[162] Bhagwat A.S., Vakoc C.R. (2015). Targeting transcription factors in cancer. Trends Cancer Res..

[163] Wang Z.-Y., Chen Z. (2008). Acute promyelocytic leukemia: From highly fatal to highly curable. Blood.

[164] Ernberg I. (2015). From the first curative targeted cancer treatment to the implementation of the most extensive healthcare reform. J. Intern. Med..

[165] Kulkarni U., Mathews V. (2021). Evolving chemotherapy free regimens for acute promyelocytic leukemia. Front. Oncol..

[166] Platzbecker U., Avvisati G., Cicconi L., Thiede C., Paoloni F., Vignetti M., Ferrara F., Divona M., Albano F., Efficace F. (2017). Improved outcomes with retinoic acid and arsenic trioxide compared with retinoic acid and chemotherapy in non-high-risk acute promyelocytic leukemia: Final results of the randomized Italian-German APL0406 trial. J. Clin. Oncol..

[167] Swart L.E., Heidenreich O. (2021). The RUNX1/RUNX1T1 network: Translating insights into therapeutic options. Exp. Hematol..

[168] Yun J.W., Bae Y.K., Cho S.Y., Koo H., Kim H.-J., Nam D.-H., Kim S.-H., Chun S., Joo K.M., Park W.-Y. (2019). Elucidation of novel therapeutic targets for acute myeloid leukemias with RUNX1-RUNX1T1 fusion. Int. J. Mol. Sci..

[169] Al-Harbi S., Aljurf M., Mohty M., Almohareb F., Ahmed S.O.A. (2020). An update on the molecular pathogenesis and potential therapeutic targeting of AML with t(8;21)(q22;q22.1);RUNX1-RUNX1T1. Blood Adv..

[170] Höllein A., Nadarajah N., Meggendorfer M., Jeromin S., Kern W., Haferlach C., Haferlach T. (2019). Molecular characterization of AML with RUNX1-RUNX1T1 at diagnosis and relapse reveals net loss of co-mutations. Hemasphere.

[171] Lam K., Zhang D.-E. (2012). RUNX1 and RUNX1-ETO: Roles in hematopoiesis and leukemogenesis. Front. Biosci..

[172] Bartel Y., Grez M., Wichmann C. (2013). Interference with RUNX1/ETO leukemogenic function by cell-penetrating peptides targeting the NHR2 oligomerization domain. Biomed. Res. Int..

[173] Ptasinska A., Assi S.A., Mannari D., James S.R., Williamson D., Dunne J., Hoogenkamp M., Wu M., Care M., McNeill H. (2012). Depletion of RUNX1/ETO in t(8;21) AML cells leads to genome-wide changes in chromatin structure and transcription factor binding. Leukemia.

[174] Heidenreich O., Krauter J., Riehle H., Hadwiger P., John M., Heil G., Vornlocher H.-P., Nordheim A. (2003). AML1/MTG8 oncogene suppression by small interfering RNAs supports myeloid differentiation of t(8;21)-positive leukemic cells. Blood.

[175] Metz A., Schanda J., Grez M., Wichmann C., Gohlke H. (2013). From determinants of RUNX1/ETO tetramerization to small-molecule protein-protein interaction inhibitors targeting acute myeloid leukemia. J. Chem. Inf. Model..

[176] Sun X.-J., Wang Z., Wang L., Jiang Y., Kost N., Soong T.D., Chen W.-Y., Tang Z., Nakadai T., Elemento O. (2013). A stable transcription factor complex nucleated by oligomeric AML1-ETO controls leukaemogenesis. Nature.

[177] Schanda J., Lee C.-W., Wohlan K., Müller-Kuller U., Kunkel H., Coco I.Q.-L., Stein S., Metz A., Koch J., Lausen J. (2017). Suppression of RUNX1/ETO oncogenic activity by a small molecule inhibitor of tetramerization. Hematologica.

[178] Spirin P.V., Lebedev T.D., Orlova N.N., Gornostaeva A.S., Prokofjeva M.M., Nikitenko N.A., Dmitriev S.E., Buzdin A.A., Borisov N.M., Aliper A.M. (2014). Silencing AML1-ETO gene expression leads to simultaneous activation of both pro-apoptotic and proliferation signaling. Leukemia.

[179] Johnson D.T., Davis A.G., Zhou J.-H., Ball E.D., Zhang D.-E. (2021). MicroRNA let-7b downregulates AML1-ETO oncogene expression in t(8;21) AML by targeting its 3’UTR. Exp. Hematol. Oncol..

[180] Roudaia L., Cheney M.D., Manuylova E., Chen W., Morrow M., Park S., Lee C.-T., Kaur P., Williams O., Bushweller J.H. (2009). CBFβ is critical for AML1-ETO and TEL-AML1 activity. Blood.

[181] Illendula A., Gilmour J., Grembecka J., Tirumala V.S.S., Boulton A., Kuntimaddi A., Schmidt C., Wang L., Pulikkan J.A., Zong H. (2016). Small molecule inhibitor of CBFβ-RUNX binding for RUNX transcription factor driven cancers. EBioMedicine.

[182] Kellaway S., Chin P.S., Barneh F., Bonifer C., Heidenreich O. (2020). t(8;21) acute myeloid leukemia as a paradigm for the understanding of leukemogenesis at the level of gene regulation and chromatin programming. Cells.

[183] Klisovic M.I., Maghraby E.A., Parthun M.R., Guimond M., Sklenar A.R., Whitman S.P., Chan K.K., Murphy T., Anon J., Archer K.J. (2003). Depsipeptide (FR 901228) promotes histone acetylation, gene transcription, apoptosis and its activity is enhanced by DNA methyltransferase inhibitors in AML1/ETO-positive leukemic cells. Leukemia.

[184] Duque-Afonso J., Yalcin A., Berg T., Abdelkarim M., Heidenreich O., Lübbert M. (2011). The HDAC class I-specific inhibitor entinostat (MS-275) effectively relieves epigenetic silencing of the LAT2 gene mediated by AML1/ETO. Oncogene.

[185] Muñoz L., Nomdedéu J.F., Villamor N., Guardia R., Colomer D., Ribera J.M., Torres J.P., Berlanga J.J., Fernández C., Llorente A. (2003). Acute myeloid leukemia with MLL rearrangements: Clinicobiological features, prognostic impact and value of flow cytometry in the detection of residual leukemic cells. Leukemia.

[186] Xu J., Li L., Xiong J., denDekker A., Ye A., Karatas H., Liu L., Wang H., Qin Z.S., Wang S. (2016). MLL1 and MLL1 fusion proteins have distinct functions in regulating leukemic transcription program. Cell Discov..

[187] Meyer C., Burmeister T., Gröger D., Tsaur G., Fechina L., Renneville A., Sutton R., Venn N.C., Emerenciano M., Pombo-de-Oliveira M.S. (2018). The MLL recombinome of acute leukemias in 2017. Leukemia.

[188] Bill M., Mrózek K., Kohlschmidt J., Eisfeld A.-K., Walker C.J., Nicolet D., Papaioannou D., Blachly J.S., Orwick S., Carroll A.J. (2020). Mutational landscape and clinical outcome of patients with de novo acute myeloid leukemia and rearrangements involving 11q23/KMT2A. Proc. Natl. Acad. Sci. USA.

[189] Liu H., Cheng E.H.Y., Hsieh J.J.D. (2009). MLL fusions: Pathways to leukemia. Cancer Biol. Ther..

[190] Argiropoulos B., Humphries R.K. (2007). Hox genes in hematopoiesis and leukemogenesis. Oncogene.

[191] Rao R.C., Dou Y. (2015). Hijacked in cancer: The KMT2 (MLL) family of methyltransferases. Nat. Rev. Cancer.

[192] Hughes C.M., Rozenblatt-Rosen O., Milne T.A., Copeland T.D., Levine S.S., Lee J.C., Hayes D.N., Shanmugam K.S., Bhattacharjee A., Biondi C.A. (2004). Menin associates with a trithorax family histone methyltransferase complex and with the hoxc8 locus. Mol. Cell.

[193] Yokoyama A., Somervaille T.C.P., Smith K.S., Rozenblatt-Rosen O., Meyerson M., Cleary M.L. (2005). The menin tumor suppressor protein is an essential oncogenic cofactor for MLL-associated leukemogenesis. Cell.

[194] Muntean A.G., Tan J., Sitwala K., Huang Y., Bronstein J., Connelly J.A., Basrur V., Elenitoba-Johnson K.S.J., Hess J.L. (2010). The PAF complex synergizes with MLL fusion proteins at HOX loci to promote leukemogenesis. Cancer Cell.

[195] Cierpicki T., Risner L.E., Grembecka J., Lukasik S.M., Popovic R., Omonkowska M., Shultis D.D., Zeleznik-Le N.J., Bushweller J.H. (2010). Structure of the MLL CXXC domain-DNA complex and its functional role in MLL-AF9 leukemia. Nat. Struct. Mol. Biol..

[196] Krivtsov A.V., Armstrong S.A. (2007). MLL translocations, histone modifications and leukaemia stem-cell development. Nat. Rev. Cancer.

[197] Kühn M.W.M., Song E., Feng Z., Sinha A., Chen C.-W., Deshpande A.J., Cusan M., Farnoud N., Mupo A., Grove C. (2016). Targeting chromatin regulators inhibits leukemogenic gene expression in NPM1 mutant leukemia. Cancer Discov..

[198] Grembecka J., He S., Shi A., Purohit T., Muntean A.G., Sorenson R.J., Showalter H.D., Murai M.J., Belcher A.M., Hartley T. (2012). Menin-MLL inhibitors reverse oncogenic activity of MLL fusion proteins in leukemia. Nat. Chem. Biol..

[199] Borkin D., He S., Miao H., Kempinska K., Pollock J., Chase J., Purohit T., Malik B., Zhao T., Wang J. (2015). Pharmacologic inhibition of the menin-MLL interaction blocks progression of MLL leukemia in vivo. Cancer Cell.

[200] Borkin D., Pollock J., Kempinska K., Purohit T., Li X., Wen B., Zhao T., Miao H., Shukla S., He M. (2016). Property focused structure-based optimization of small molecule inhibitors of the protein-protein interaction between menin and mixed lineage leukemia (MLL). J. Med. Chem..

[201] Borkin D., Klossowski S., Pollock J., Miao H., Linhares B.M., Kempinska K., Jin Z., Purohit T., Wen B., He M. (2018). Complexity of blocking bivalent protein-protein interactions: Development of a highly potent inhibitor of the menin-mixed-lineage leukemia interaction. J. Med. Chem..

[202] Klossowski S., Miao H., Kempinska K., Wu T., Purohit T., Kim E., Linhares B.M., Chen D., Jih G., Perkey E. (2020). Menin inhibitor MI-3454 induces remission in MLL1-rearranged and NPM1-mutated models of leukemia. J. Clin. Investig..

[203] Bushweller J.H. (2019). Targeting transcription factors in cancer—From undruggable to reality. Nat. Rev. Cancer.

[204] Liu P., Tarlé S.A., Hajra A., Claxton D.F., Marlton P., Freedman M., Siciliano M.J., Collins F.S. (1993). Fusion between transcription factor CBF beta/PEBP2 beta and a myosin heavy chain in acute myeloid leukemia. Science.

[205] Kamikubo Y., Zhao L., Wunderlich M., Corpora T., Hyde R.K., Paul T.A., Kundu M., Garrett L., Compton S., Huang G. (2010). Accelerated leukemogenesis by truncated CBFβ-SMMHC defective in high-affinity binding with RUNX1. Cancer Cell.

[206] Mandoli A., Singh A.A., Jansen P.W.T.C., Wierenga A.T.J., Riahi H., Franci G., Prange K., Saeed S., Vellenga E., Vermeulen M. (2014). CBFB-MYH11/RUNX1 together with a compendium of hematopoietic regulators, chromatin modifiers and basal transcription factors occupies self-renewal genes in inv(16) acute myeloid leukemia. Leukemia.

[207] Castilla L.H., Wijmenga C., Wang Q., Stacy T., Speck N.A., Eckhaus M., Marín-Padilla M., Collins F.S., Wynshaw-Boris A., Liu P.P. (1996). Failure of embryonic hematopoiesis and lethal hemorrhages in mouse embryos heterozygous for a knocked-in leukemia gene CBFB-MYH11. Cell.

[208] Okuda T., van Deursen J., Hiebert S.W., Grosveld G., Downing J.R. (1996). AML1, the target of multiple chromosomal translocations in human leukemia, is essential for normal fetal liver hematopoiesis. Cell.

[209] Wang Q., Stacy T., Miller J.D., Lewis A.F., Gu T.L., Huang X., Bushweller J.H., Bories J.C., Alt F.W., Ryan G. (1996). The CBFbeta subunit is essential for CBFalpha2 (AML1) function in vivo. Cell.

[210] Castilla L.H., Garrett L., Adya N., Orlic D., Dutra A., Anderson S., Owens J., Eckhaus M., Bodine D., Liu P.P. (1999). The fusion gene Cbfb-MYH11 blocks myeloid differentiation and predisposes mice to acute myelomonocytic leukaemia. Nat. Genet..

[211] Castilla L.H., Perrat P., Martinez N.J., Landrette S.F., Keys R., Oikemus S., Flanegan J., Heilman S., Garrett L., Dutra A. (2004). Identification of genes that synergize with Cbfb-MYH11 in the pathogenesis of acute myeloid leukemia. Proc. Natl. Acad. Sci. USA.

[212] Opatz S., Bamopoulos S.A., Metzeler K.H., Herold T., Ksienzyk B., Bräundl K., Tschuri S., Vosberg S., Konstandin N.P., Wang C. (2020). The clinical mutatome of core binding factor leukemia. Leukemia.

[213] Zhen T., Cao Y., Ren G., Zhao L., Hyde R.K., Lopez G., Feng D., Alemu L., Zhao K., Liu P.P. (2020). RUNX1 and CBFβ-SMMHC transactivate target genes together in abnormal myeloid progenitors for leukemia development. Blood.

[214] Illendula A., Pulikkan J.A., Zong H., Grembecka J., Xue L., Sen S., Zhou Y., Boulton A., Kuntimaddi A., Gao Y. (2015). A small-molecule inhibitor of the aberrant transcription factor CBFβ-SMMHC delays leukemia in mice. Science.

[215] Pulikkan J.A., Hegde M., Ahmad H.M., Belaghzal H., Illendula A., Yu J., O’Hagan K., Ou J., Muller-Tidow C., Wolfe S.A. (2018). CBFβ-SMMHC inhibition triggers apoptosis by disrupting MYC chromatin dynamics in acute myeloid leukemia. Cell.

[216] Richter L.E., Wang Y., Becker M.E., Coburn R.A., Williams J.T., Amador C., Hyde R.K. (2019). HDAC1 is a required cofactor of CBFβ-SMMHC and a potential therapeutic target in inversion 16 acute myeloid leukemia. Mol. Cancer Res..

[217] Biernacki M.A., Foster K.A., Woodward K.B., Coon M.E., Cummings C., Cunningham T.M., Dossa R.G., Brault M., Stokke J., Olsen T.M. (2020). CBFB-MYH11 fusion neoantigen enables T cell recognition and killing of acute myeloid leukemia. J. Clin. Investig..

[218] Wong S., Witte O.N. (2004). The BCR-ABL story: Bench to bedside and back. Annu. Rev. Immunol..

[219] Rossari F., Minutolo F., Orciuolo E. (2018). Past, present, and future of Bcr-Abl inhibitors: From chemical development to clinical efficacy. J. Hematol. Oncol..

[220] O’Hare T., Deininger M.W.N., Eide C.A., Clackson T., Druker B.J. (2011). Targeting the BCR-ABL signaling pathway in therapy-resistant Philadelphia chromosome-positive leukemia. Clin. Cancer Res..

[221] Eide C.A., Zabriskie M.S., Savage Stevens S.L., Antelope O., Vellore N.A., Than H., Schultz A.R., Clair P., Bowler A.D., Pomicter A.D. (2019). Combining the allosteric inhibitor asciminib with ponatinib suppresses emergence of and restores efficacy against highly resistant BCR-ABL1 mutants. Cancer Cell.

[222] Hughes T.P., Mauro M.J., Cortes J.E., Minami H., Rea D., DeAngelo D.J., Breccia M., Goh Y.-T., Talpaz M., Hochhaus A. (2019). Asciminib in chronic myeloid leukemia after ABL kinase inhibitor failure. N. Engl. J. Med..

[223] Garcia-Gutiérrez V., Luna A., Alonso-Dominguez J.M., Estrada N., Boque C., Xicoy B., Giraldo P., Angona A., Alvarez-Larrán A., Sanchez-Guijo F. (2021). Safety and efficacy of asciminib treatment in chronic myeloid leukemia patients in real-life clinical practice. Blood Cancer J..

[224] Hoch M., Sato M., Zack J., Quinlan M., Sengupta T., Allepuz A., Aimone P., Hourcade-Potelleret F. (2021). Pharmacokinetics of asciminib in individuals with hepatic or renal impairment. J. Clin. Pharmacol..

[225] Li S., Couvillon A.D., Brasher B.B., Van Etten R.A. (2001). Tyrosine phosphorylation of Grb2 by Bcr/Abl and epidermal growth factor receptor: A novel regulatory mechanism for tyrosine kinase signaling. EMBO J..

[226] Sattler M., Mohi M.G., Pride Y.B., Quinnan L.R., Malouf N.A., Podar K., Gesbert F., Iwasaki H., Li S., Van Etten R.A. (2002). Critical role for Gab2 in transformation by BCR/ABL. Cancer Cell.

[227] Chu S., Li L., Singh H., Bhatia R. (2007). BCR-tyrosine 177 plays an essential role in Ras and Akt activation and in human hematopoietic progenitor transformation in chronic myelogenous leukemia. Cancer Res..

[228] Naka K., Hoshii T., Muraguchi T., Tadokoro Y., Ooshio T., Kondo Y., Nakao S., Motoyama N., Hirao A. (2010). TGF-β-FOXO signalling maintains leukaemia-initiating cells in chronic myeloid leukaemia. Nature.

[229] Agarwal A., Bumm T.G.P., Corbin A.S., O’Hare T., Loriaux M., VanDyke J., Willis S.G., Deininger J., Nakayama K.I., Druker B.J. (2008). Absence of SKP2 expression attenuates BCR-ABL-induced myeloproliferative disease. Blood.

[230] Markova B., Albers C., Breitenbuecher F., Melo J.V., Brümmendorf T.H., Heidel F., Lipka D., Duyster J., Huber C., Fischer T. (2010). Novel pathway in Bcr-Abl signal transduction involves Akt-independent, PLC-γ1-driven activation of mTOR/p70S6-kinase pathway. Oncogene.

[231] Xie S., Wang Y., Liu J., Sun T., Wilson M.B., Smithgall T.E., Arlinghaus R.B. (2001). Involvement of Jak2 tyrosine phosphorylation in Bcr-Abl transformation. Oncogene.

[232] Klejman A., Schreiner S.J., Nieborowska-Skorska M., Slupianek A., Wilson M., Smithgall T.E., Skorski T. (2002). The Src family kinase Hck couples BCR/ABL to STAT5 activation in myeloid leukemia cells. EMBO J..

[233] Schafranek L., Nievergall E., Powell J.A., Hiwase D.K., Leclercq T., Hughes T.P., White D.L. (2015). Sustained inhibition of STAT5, but not JAK2, is essential for TKI-induced cell death in chronic myeloid leukemia. Leukemia.

[234] Berger A., Sexl V., Valent P., Moriggl R. (2014). Inhibition of STAT5: A therapeutic option in BCR-ABL1-driven leukemia. Oncotarget.

[235] Page B.D.G., Khoury H., Laister R.C., Fletcher S., Vellozo M., Manzoli A., Yue P., Turkson J., Minden M.D., Gunning P.T. (2012). Small molecule STAT5-SH2 domain inhibitors exhibit potent antileukemia activity. J. Med. Chem..

[236] Nelson E.A., Walker S.R., Weisberg E., Bar-Natan M., Barrett R., Gashin L.B., Terrell S., Klitgaard J.L., Santo L., Addorio M.R. (2011). The STAT5 inhibitor pimozide decreases survival of chronic myelogenous leukemia cells resistant to kinase inhibitors. Blood.

[237] He Y., Khan S., Huo Z., Lv D., Zhang X., Liu X., Yuan Y., Hromas R., Xu M., Zheng G. (2020). Proteolysis targeting chimeras (PROTACs) are emerging therapeutics for hematologic malignancies. J. Hematol. Oncol..

[238] Rohrbacher M., Hasford J., Wiernik P.H., Dutcher J.P., Gertz M.A. (2018). Epidemiology and Etiology of Chronic Myeloid Leukemia. Neoplastic Diseases of the Blood.

[239] Shallis R.M., Wang R., Davidoff A., Ma X., Zeidan A.M. (2019). Epidemiology of acute myeloid leukemia: Recent progress and enduring challenges. Blood Rev..

[240] Guo L.-M., Xi J.-S., Ma Y., Shao L., Nie C.-L., Wang G.-J. (2014). ARID5B gene rs10821936 polymorphism is associated with childhood acute lymphoblastic leukemia: A meta-analysis based on 39,116 subjects. Tumour Biol..

[241] Gurnari C., Pagliuca S., Visconte V. (2020). Deciphering the therapeutic resistance in acute myeloid leukemia. Int. J. Mol. Sci..

[242] Guo L., Li J., Zeng H., Guzman A.G., Li T., Lee M., Zhou Y., Goodell M.A., Stephan C., Davies P.J.A. (2020). A combination strategy targeting enhancer plasticity exerts synergistic lethality against BETi-resistant leukemia cells. Nat. Commun..

[243] Knoechel B., Roderick J.E., Williamson K.E., Zhu J., Lohr J.G., Cotton M.J., Gillespie S.M., Fernandez D., Ku M., Wang H. (2014). An epigenetic mechanism of resistance to targeted therapy in T cell acute lymphoblastic leukemia. Nat. Genet..

[244] Domingues A.F., Kulkarni R., Giotopoulos G., Gupta S., Vinnenberg L., Arede L., Foerner E., Khalili M., Adao R.R., Johns A. (2020). Loss of Kat2a enhances transcriptional noise and depletes acute myeloid leukemia stem-like cells. eLife.

[245] Shlush L.I., Mitchell A., Heisler L., Abelson S., Ng S.W.K., Trotman-Grant A., Medeiros J.J.F., Rao-Bhatia A., Jaciw-Zurakowsky I., Marke R. (2017). Tracing the origins of relapse in acute myeloid leukaemia to stem cells. Nature.

[246] Gentles A.J., Plevritis S.K., Majeti R., Alizadeh A.A. (2010). Association of a leukemic stem cell gene expression signature with clinical outcomes in acute myeloid leukemia. JAMA.

